# Novel alternative ribonucleotide excision repair pathways in human cells by DDX3X and specialized DNA polymerases

**DOI:** 10.1093/nar/gkaa948

**Published:** 2020-11-02

**Authors:** Valentina Riva, Anna Garbelli, Federica Casiraghi, Francesca Arena, Claudia Immacolata Trivisani, Assunta Gagliardi, Luca Bini, Martina Schroeder, Antonio Maffia, Simone Sabbioneda, Giovanni Maga

**Affiliations:** Institute of Molecular Genetics IGM-CNR ‘Luigi Luca Cavalli-Sforza’, via Abbiategrasso 207, I-27100 Pavia, Italy; Institute of Molecular Genetics IGM-CNR ‘Luigi Luca Cavalli-Sforza’, via Abbiategrasso 207, I-27100 Pavia, Italy; Institute of Molecular Genetics IGM-CNR ‘Luigi Luca Cavalli-Sforza’, via Abbiategrasso 207, I-27100 Pavia, Italy; Institute of Molecular Genetics IGM-CNR ‘Luigi Luca Cavalli-Sforza’, via Abbiategrasso 207, I-27100 Pavia, Italy; Department of Biotechnology, Chemistry and Pharmacy, University of Siena, Via A. De Gasperi 2, I-53100 Siena, Italy; Department of Life Sciences, Via A. Moro 2, University of Siena, I-53100 Siena, Italy; Department of Life Sciences, Via A. Moro 2, University of Siena, I-53100 Siena, Italy; Kathleen Lonsdale Institute for Human Health Research, Biology Department, Maynooth University, Maynooth, Co. Kildare, Ireland; Institute of Molecular Genetics IGM-CNR ‘Luigi Luca Cavalli-Sforza’, via Abbiategrasso 207, I-27100 Pavia, Italy; Institute of Molecular Genetics IGM-CNR ‘Luigi Luca Cavalli-Sforza’, via Abbiategrasso 207, I-27100 Pavia, Italy; Institute of Molecular Genetics IGM-CNR ‘Luigi Luca Cavalli-Sforza’, via Abbiategrasso 207, I-27100 Pavia, Italy

## Abstract

Removal of ribonucleotides (rNMPs) incorporated into the genome by the ribonucleotide excision repair (RER) is essential to avoid genetic instability. In eukaryotes, the RNaseH2 is the only known enzyme able to incise 5′ of the rNMP, starting the RER process, which is subsequently carried out by replicative DNA polymerases (Pols) δ or ϵ, together with Flap endonuclease 1 (Fen-1) and DNA ligase 1. Here, we show that the DEAD-box RNA helicase DDX3X has RNaseH2-like activity and can support fully reconstituted *in vitro* RER reactions, not only with Pol δ but also with the repair Pols β and λ. Silencing of DDX3X causes accumulation of rNMPs in the cellular genome. These results support the existence of alternative RER pathways conferring high flexibility to human cells in responding to the threat posed by rNMPs incorporation.

## INTRODUCTION

Compelling experimental evidence accumulating in the last years, led to the realization that the most abundant type of endogenous DNA damage in mammalian cells is represented by the >1 million ribonucleotides (rNMPs) erroneously incorporated into the genome at each round of replication. Unexpectedly, the number of erroneously incorporated rNMPs is even higher than the well-known oxidized bases or cyclobutane pyrimidine dimers (1). rNMPs misincorporation leads to deleterious consequences: their presence into the genome makes the sugar-phosphate backbone ∼100 000- times more prone to hydrolysis due to the presence of the 2′-OH in their ribose ring. This might lead to DNA breaks and replication fork arrest. In addition, the structural modification imposed by the presence of rNMPs, could affect protein-DNA interactions, nucleosome structure and the activity of DNA-processing enzymes such as topoisomerases and ligases (2). Indeed, accumulation of rNMPs in the genome has been linked to increased mutagenesis and genetic instability in eukaryotic cells (3).

The only enzymes known so far able to remove rNMPs into a DNA stretch are RNaseH-type enzymes. There are two main classes of RNaseH enzymes: RNaseH1 and RNaseH2 that display significant differences in their enzymatic properties. RNaseH1 normally removes DNA:RNA hybrids (or R-loops) of at least three-four residues. In contrast, RNaseH2 is the only enzyme able to process single rNMPs into a dsDNA context cleaving on the 5′ side of them and promoting an error-free and specialized repair pathway called ribonucleotide excision repair (RER) (4). After RNaseH2-dependent cleavage at the 5′-side of the rNMP creates a nick, the 3′-OH end generated by the incision is elongated by the replicative holoenzyme constituted by DNA polymerase (Pol) δ or ϵ, PCNA and RF-C. Through strand displacement synthesis, a 5′-rNMP terminated flap is generated, which is cut by the nuclease Fen-1 and an intact DNA strand without rNMPs is reconstituted by DNA ligase 1 (5). So far, the entire RER pathway was reconstituted *in vitro* thanks to yeast enzymes and no other Pol has been shown, up to now, to be able to act in RER reactions (6).

In RNaseH2-deficient mammalian cells, there is the activation of an alternative and highly mutagenic pathway directed by Top-1 enzyme that is deleterious for genome stability (7,8). Moreover, in humans the inactivation of RNaseH2 causes the Aicardi-Goutier syndrome (AGS), a rare genetic neurological disorder characterized by inflammatory response activation in the absence of exogenous stimuli (4).

The DEAD-box RNA helicase DDX3X is a well-known enzyme for its involvement and therapeutic relevance in viral infections and cancer (9). It operates in all the aspects of RNA metabolism in addition to cell proliferation pathways and innate immunity (9,10). DDX3X loss promotes tumorigenesis (11), neurodegenerative diseases (12) and triggers DNA damage and embryonic lethality in mice (13). More important, recent evidence showed through chromatin immunoprecipitation analysis also a possible role of DDX3X in genome stability maintenance: DDX3X binds promoter regions regulating the expression of two DNA repair factors, DDB2 and XPA (10). Published data have implicated DDX3X in the resolution of DNA:RNA hybrids and RNA secondary structures, as well as in the degradation of RNAs (14).

Different studies utilized specifically mutated polymerases to increase the level of misincorporated rNMPs in the genome. Comparing cells with wild type- and deficient- RNaseH2 enzyme, it appears clear that it is not just the absence of RNaseH2, but also of the ribonucleotide processing that causes deleterious consequences. If the level of rNMPs exceeds a specific threshold, there is the insurgence of cellular stresses also in the presence of a completely functional RNaseH2 (4). Moreover, in prokaryotes the absence of both RNaseH1 and 2 in mutant strains causes a level of spontaneous mutagenesis that is lower than expected. This means that there are probably some ‘backup enzymes’ into the cell, less effective than RNaseH2, but still able to maintain partial genome integrity (6).

Thanks to biochemical *in vitro* characterization and preliminary *in vivo* evidence, here we show that the RNA helicase DDX3X has RNaseH2-like activity able to initiate RER in *in vitro* reconstituted systems and that its silencing causes accumulation of rNMPs in cellular genome. Moreover, we demonstrate that the specialized DNA repair Pols β and λ can fully support *in vitro* reconstituted RER reactions, with either RNaseH2 or DDX3X. These results support the existence of alternative RER pathways in human cells, suggesting a hitherto unappreciated flexibility of cells in responding to the threat posed by rNMPs incorporation.

## MATERIALS AND METHODS

### Chemicals

Unlabelled rNMPs and dNTPs were from Roche Molecular Biochemicals (Mannheim, Germany). All other reagents were of analytical grade and were purchased from Merck (Darmstadt, Germany) or Fluka (Munich, Germany).

### Nucleic acids substrates

Oligonucleotides were purchased from Biomers.net GmbH (Ulm, Germany) or Metabion AG (Steinkirchen, Germany). The sequences of the substrates used are (bold letters indicate ribonucleotides):

ds 55rC/55 mer Substrate *D_39_R_1_D_15_: D_55_
5′-CCAACACACAACACCGTGTGAATTCGGCACTGGCCGTCGTATGCTCTTGGTTGTA-3′
3′-GGTTGTGTGTTGTGGCACACTTAAGCCGTGACCGGCAG**C**ATACGAGAACCAACAT-5′FAM
ds 24rC/24mer Substrate *D_19_R_1_D_4_: D_24_
5′-CTACGGCTCACACTATCTCACACT-3′
3′-GATG**C**CGAGTGTGATAGAGTGTGA-5′FAM
ds 24rC/24mer Substrate *D_18_R_1_D_5_: D_24_
5′-CTACGGCTCACACTATCTCACACT-3′
3′-GATGC**C**GAGTGTGATAGAGTGTGA-5′FAM
ds 41rCGUA/41 mer Substrate *D_19_R_4_D_18_: D_41_
5′-TATCCACCAATACTACCCTACGCACACCTCCACTCAAACTC-3′
3′-ATAGGTGGTTATGATGGG**AUGC**GTGTGGAGGTGAGTTTGAG-5′FAM
ds 18/38 mer RNA Substrate *R_18_: R_38_
5′-AUGAAGGUUUGAGUUGAGUGGAGAUAGUGGAGGGUAGU-3′
3′-UACUUCCAAACUCAACUC-5′ FAM
FEN-1 Flap control Substrate D_54_: *D_48_ + D_30_
54mer:
5′-TACCAACAACTTTCAACCCCAATCTACCCCTCTTCCACCCAATTCCATTATACC-3′
30mer:
5′-GGTATAATGGAATTGGGTGGAAGAGGGGTA-3′
48mer(flap):
FAM 5′-TACCTTAAAACATATTCTCTAACTGATTGGGGTTGAAAGTTGTTGGTA-3′
Single- and double- underlined sequences indicate the hybridization positions of the respective oligonucleotides, to generate a dsDNA with a 24 nucleotides (nt) flap.
ds 55rC/55oxoG Substrate *D_39_R_1_D_15_: D_39_8oxoG_1_D_15_
5′ -CCAACACACAACACCGTGTGAATTCGGCACTGGCCGTC**_8_**TATGCTCTTGGTTGTA- 3′
3′ -GGTTGTGTGTTGTGGCACACTTAAGCCGTGACCGGCAG**C**ATACGAGAACCAACAT-5′FAM

Each 6-carboxyfluorescein (FAM) 5′-labeled primer was mixed to the complementary template oligonucleotide at 1:1 (M/M) ratio in the presence of 150 mM HEPES–KOH pH 7.4, 500 mM KCl, 10 mM MgCl_2_ and 250 mM NH_4_Ac, heated at 95°C for 5 min and then slowly cooled down at room temperature.

### Proteins production and purification

Human recombinant full length DDX3X was cloned in the *E. coli* expression vector pET-30a(+). ShuffleT7 *E. coli* cells were transformed with the plasmid and grown at 37°C up to OD_600_ = 0.7. DDX3X expression was induced with 0.5 mM IPTG at 15°C O/N. Cells were harvested by centrifugation, lysed and the crude extract centrifuged at 100 000×g for 60 min at 4°C in a Beckman centrifuge before being loaded onto a FPLC Ni-NTA column from GE Healthcare (Uppsala, Sweden). Column was equilibrated in Buffer A (50 mM Tris–HCl pH 8.0, 250 mM NaCl, 25 mM Imidazole and 20% glycerol). After extensive washing in Buffer A, the column was eluted with a linear gradient in Buffer A from 25 to 250 mM Imidazole. Proteins in the eluted fractions were visualized on SDS-PAGE and tested for the presence of DDX3X by Western Blot with anti-DDX3X A300-A474 from BETHYL (Montgomery, United States) at 1:4000 dilution in 5% milk. Fractions containing the purest DDX3X protein were pooled and dialyzed using Slide-A-Lyzer^®^ MINI Dialysis Devices 20K MWCO from Thermo Scientific (Rockford, United States) for 3 h (25 mM Tris–HCl pH 8.0, 0.5 mM DTT, 100 mM NaCl, glycerol 20%) in order to remove the salt excess. Finally, proteins were stored at –80°C.

Human recombinant DDX3X mutants here specified were induced and purified as the full-length protein (E256A/R262A, R351A, D347A/D350A). E256A and R262A mutants were cloned in the *Escherichia coli* expression vector pET-30a(+) and BL21(DE3) cells were transformed with the plasmids. Proteins induction and purification were the same as for the full-length protein, with the only difference that proteins were not dialyzed to avoid further loss of material due to the dialysis procedure.

Human recombinant DDX3XΔins (1–248/260–662) was cloned in the *E. coli* expression vector pTrchisA. BL21 *E. coli* cells were transformed with the plasmid and grown at 37°C up to OD_600_ = 1. DDX3X expression was induced with 0.5 mM IPTG at 15°C O/N. Purification procedure was the same of full-length protein.

Human recombinant DDX3X (132–607) truncated mutant was expressed and purified as previously described (15). Human recombinant Pols λ and β (16), Pol δ (17) and RNaseH2 (18) were expressed and purified as described. Human Fen-1 and Human DNA ligase 1 were both purchased from Origene (Rockville, United States). Human recombinant full length DDX5 was cloned in the *E. coli* expression vector pET-30a(+). BL21(DE3) *E. coli* cells were transformed with the plasmid and grown at 37°C up to OD_600_ = 0.7. DDX5 expression was induced with 0.5 mM IPTG at 37°C for 4 h. Cells were harvested by centrifugation and lysed and the crude extract centrifuged at 13 000×g for 60 min at 4°C in a Beckman centrifuge before being loaded onto Dyanabeads^®^ His-Tag Isolation & Pulldown beads from Life Technologies (Rockford, United States). Protein was eluted according to the manual.

### Proteomic analysis

*2-D electrophoresis (2-DE) and mass spectrometry*. The first electrophoresis run (IsoElectroFocusing, IEF) was performed on 18 cm long, non-linear pH 3–10 gradient Immobiline-polyacrylamide DryStrip gels (IPG strips; GE Healthcare) using the Ettan™ IPGphor system (GE Healthcare). For analytical and MS-preparative gels, strips were rehydrated with 20 and 60 μg of protein, respectively, denatured in 350 μl of 2-DE buffer [8M urea, 4% (w/v) CHAPS, and 1% (w/v) dithioerythritol (DTE)] and 0.2% or 2% (v/v) IPG-buffer (pH 3–10; GE Healthcare), for 1 h at 0 V and overnight at 30 V, at 16°C. IEF was performed at 16°C and complete protein focusing was reached when a total of 100 000 Vh was applied. The focused strips were equilibrated for 12 min in 6 M urea, 30% (v/v) glycerol, 2% (w/v) SDS, 0.05 M Tris–HCl pH 6.8, 2% (w/v) DTE; and for further 5 min in 6 M urea, 30% (v/v) glycerol, 2% (w/v) SDS, 0.05 M Tris–HCl pH 6.8, 2.5% (w/v) iodoacetamide, and trace of bromophenol blue. The second dimension (SDS-PAGE) was run on house-made 9–16% polyacrylamide linear gradient gels. at 10°C, in Protean II Multi Cell (Bio-Rad Laboratories, Hercules, CA, USA) applying 40 mA/gel constant current. The run was stopped when the bromophenol blue dye reached the end of the gel. Analytical and MS-preparative gels were stained with ammoniacal silver nitrate and with a MS-compatible silver staining, respectively. 2-DE stained gels were scanned using the ImageScanner III (GE Healthcare), and the acquired images were analyzed by ImageMaster 2D Platinum v6.0 software (GE Healthcare).

Protein spots cut from MS-preparative gels, were destained and acetonitrile dehydrated. Spots were rehydrated in trypsin solution (Sigma, St. Louis, United States) and in-gel trypsin digested overnight at 37°C. 1.25 μl of each tryptic peptides sample was directly spotted onto the MALDI target and air-dried. Then 0.75 μl of matrix solution (a saturated solution of alpha-cyano-4-hydroxycynnamic acid in 50% (v/v) acetonitrile and 0.5% (v/v) trifluoroacetic acid) was added to the dried samples and allowed to dry again. Protein identification was carried out by peptide mass fingerprinting (PMF) using an Ultraflex III MALDI-TOF/TOF mass spectrometer (Bruker Daltonics, Billerica, USA). Mass spectra were acquired with the mass spectrometer in reflector positive mode with a laser frequency of 100 Hz and analyzed by Flex Analysis software v.3.0. PMF database searching was carried out in SwissProt database (version 2018_01; 556568 sequences; 199530821 residues) using the on-line available Mascot software (Matrix Science Ltd., London, UK, http://www.matrixscience.com). The following database search criteria were set: *E. coli* or *Homo sapiens* as taxonomy, 100 ppm as peptide tolerance, trypsin as the digestion enzyme with one allowed missed cleavage, cysteine carbamidomethylation as fixed modification, and methionine oxidation as variable modification. For protein identifications the number of matched peptides, the extent of sequence coverage, and the probabilistic score were considered.

#### Liquid chromatography–tandem MS (LC–MS/MS) analysis and protein identification

Entire gel lanes were processed with STAGE-diging protocol as described (19) and peptides were resuspended in 10 μl of solvent A. 3 μl were injected on a quadrupole Orbitrap Q-Exactive mass spectrometer (Thermo Scientific) coupled with an UHPLC Easy-nLC 1000 (Thermo Scientific) with a 25 cm fused-silica emitter of 75 μm inner diameter. Columns were packed in-house with ReproSil-Pur C18-AQ beads (Dr Maisch Gmbh, Ammerbuch, Germany), 1.9 μm of diameter, using a high-pressure bomb loader (Proxeon, Odense, Denmark).

Peptides separation was achieved with a linear gradient from 95% solvent A (2% ACN, 0.1% formic acid) to 40% solvent B (80% acetonitrile, 0.1% formic acid) over 30 min and from 40% to 100% solvent B in 2 min at a constant flow rate of 0.25 μL/min, with a single run time of 33 min in case of single bands, or with a linear gradient from 95% solvent A to 40% solvent B over 55 min and from 40% to 100% solvent B in 5 min at a constant flow rate of 0.25 μl/min for peptides obtained with STAGE-diging.

MS data were acquired using a data-dependent top 12 method, the survey full scan MS spectra (300–1750 Th) were acquired in the Orbitrap with 70 000 resolution, AGC target 1e6, IT 120 ms. For HCD spectra resolution was set to 35000, AGC target 1e5, IT 120 ms; normalized collision energy 25% and isolation width of 3.0 *m*/*z*.

For protein identification the raw data were processed using Proteome Discoverer (version 1.4.0.288, Thermo Fischer Scientific). MS (2) spectra were searched with Mascot engine against Uniprot_CP_HomoSapiens_20170110 database (unknown version, 156576 entries) and the Uniprot_Swissprot_all_20170110 database (selected for *E. coli*, unknown version, 23014 entries), with the following parameters: enzyme Trypsin, maximum missed cleavage 2, fixed modification carbamidomethylation (C), variable modification oxidation (M) and protein N-terminal acetylation, peptide tolerance 10 ppm, MS/MS tolerance 20 mmu. Peptide Spectral Matches (PSM) were filtered using percolator based on *q*-values at a 0.01 FDR (high confidence). Proteins were considered identified with 2 unique high confident peptides (20). Scaffold (version Scaffold_4.3.4, Proteome Software Inc., Portland, OR) was used to validate MS/MS based peptide and protein identifications. Peptide identifications were accepted if they could be established at >95.0% probability by the Peptide Prophet algorithm (21) with Scaffold delta-mass correction. Protein identifications were accepted if they could be established at >99.0% probability and contained at least 3 identified peptides. Protein probabilities were assigned by the Protein Prophet algorithm (22). Proteins that contained similar peptides and could not be differentiated based on MS/MS analysis alone were grouped to satisfy the principles of parsimony. Proteins sharing significant peptide evidence were grouped into clusters.

### Nuclease assays

The nuclease activity of both human RNaseH2 and DDX3X enzymes was monitored on a double stranded (ds) DNA bearing a single ribonucleotide in the strand labelled at the 5′-end with a 6-FAM fluorescent group. A final concentration of 50 nM DNA substrate was used in the experiments. The amounts of RNaseH2 and DDX3X proteins are indicated in the respective figure legends. Reactions were performed in 20 mM Tris–HCl pH 8.8, 10 mM (NH_4_)_2_SO_4_, 10 mM KCl, 2 mM MgSO_4_, 0.1% TritonX-100 at 37°C for 60 min (unless specified in figure legends). Finally, the reaction mixtures were stopped by addition of standard denaturing gel loading buffer (95% formamide, 10 mM EDTA, xylene cyanol and bromophenol blue), heated at 95°C for 5 min and loaded on 7 M urea 12% polyacrylamide (PAA) gel, run at 40 W for 2 h in TBE buffer. Substrates and products were quantified by laser scanning densitometry (Typhoon-TRIO, GE Healthcare).

### Non denaturing gel-based helicase assays

The helicase activity of DDX3X was monitored by measuring the conversion of a double stranded (ds) RNA (labeled at the 5′-end of one strand with a 6-FAM fluorescent group) into single stranded (ss) nucleic acid. A final concentration of 50 nM RNA substrate was used in the experiments, unless otherwise stated. The amounts of DDX3X proteins are indicated in the respective figure legends. Reactions were performed in 20 mM Tris–HCl pH 8, 2 mM DTT, 70 mM KCl and 1 mM ATP, 2 mM MgCl_2_ at 37°C for 30 min (unless otherwise stated in figure legends) and stopped by adding EDTA 50 mM pH 8. Products were separated through non-denaturing 7% PAGE at 5 W for 3 h in TBE buffer at 4°C.

Substrates and products were quantified by laser scanning densitometry (Typhoon-TRIO, GE Healthcare).

### Helicase assay based on fluorescence resonance energy transfer (FRET)

The FRET helicase assay was performed as described (23). Briefly, the dsRNA substrate for the helicase assay was prepared by hybridizing two ss RNA oligonucleotides with the following sequences:

- Fluo-FAM 5′-UUUUUUUUUUUUUUAGUACCGCCACCCUCAGAACC-3′
- Qu-BHQ1 5′-GGUUCUGAGGGUGGCGGUACUA-3′
- DNA capture 5′-TAGTACCGCCACCCTCAGAACC-3′

The sequence in Fluo-FAM complementary to Qu-BHQ1 is underlined. Flu-FAM carries a 6- carboxyfluorescein fluorophore at its 3′ end, while Qu-BHQ1 carries a Black Hole quencher group at its 5′ end. The DNA capture oligonucleotide is complementary to the Qu-BHQ1 oligonucleotide but bears no modifications.

Helicase assay using the dsRNA substrate was performed in 20 mM Tris-HCl pH 8, 70 mM KCl, 2 mM MgCl_2_, 2 mM dithiothreititol, 12 units RNasin (Promega, MD, USA), 2 mM ATP, 100 nM capture strand and different and increasing concentration of dsRNA in 20 μl of reaction volume. The unwinding reaction was started by adding DDX3X recombinant protein and R351A mutant and carried out at 37°C for 40 min using a LightCycler 480 (Roche). The fluorescence intensity was recorded every 30 s. Data of fluorescence signals were analyzed by linear interpolation and the corresponding slope values were used to determine the apparent unwinding rate.

### Ribonucleotide excision repair assays

The ribonucleotide excision repair (RER) assay was reconstituted *in vitro* by reproducing all steps of physiological RER: incision of a single rMNP embedded in a dsDNA by RNaseH2 or DDX3X, was followed by DNA synthesis with Pols δ, β or λ, 5′ rMNP-terminated flap removal by Fen-1 and ligation by DNA ligase 1 to generate as final product a dsDNA without embedded rMNP. A final concentration of 50 nM DNA substrate was used in the experiments. The amounts of RNaseH2, DDX3X, Pols δ and β, PCNA and dNTPs are indicated in the respective figure legends. Reactions were performed in 20 mM Tris–HCl pH 8.8, 10 mM (NH_4_)_2_SO_4_, 10 mM KCl, 2 mM MgSO_4_, 0.1% Triton X-100 at 37°C for 60 min (unless specified in the figures). To prove that no more ribonucleotides were present into the fully repaired DNA substrate, at the end of the RER reaction we inactivated all the enzymes at 75°C for 10 min. Then, we left the reaction at room temperature for 10 min and we proceeded adding 100 nM of human RNaseH2 for 45 min. The dsDNA product without embedded rMNP, will be resistant to the digestion of RNaseH2 enzyme. Finally, the reaction mixtures was stopped by addition of standard denaturing gel loading buffer (95% formamide, 10 mM EDTA, xylene cyanol and bromophenol blue), heated at 95°C for 5 min and loaded on 7 M urea 12% polyacrylamide (PAA) gel at 40 W for 2 h in TBE buffer. Substrates and products were quantified by laser scanning densitometry (Typhoon-TRIO, GE Healthcare).

### Cell extracts and DDX3X quantification

As already described (23) 10^7^ cells were ruptured with a Dounce homogenizer in Lysis Buffer (50 mM Tris–HCl pH 8.0, 0.1% SDS, 350 mM NaCl, 0.25% Triton X-100, protease inhibitor cocktail (Sigma). The lysate was incubated 30 min on ice, sonicated for 5 min (at 30 s intervals) and centrifuged at 15 000 × g for 10 min at 4°C. The protein concentration in the supernatant (crude extract) was quantified with Bradford. For DDX3X quantification, increasing concentrations of crude extract (5, 10, 20, 40 μg) were loaded on a SDS-PAGE alongside known concentrations (50 ng, 100 ng, 150 ng, 200 ng) of recombinant purified DDX3X. Separated proteins were subjected to Western Blot analysis with anti-DDX3X A300-475A (BETHYL*)* at 1:4000 dilution in 5% milk. The blot was next incubated with an HRP-conjugated secondary antibody diluted to 1:5000 and the bands corresponding to DDX3X were visualized with Enhanced Chemiluminescent Substrate (Westar Nova 2.0, Cyanagen, Bologna, Italy) using a ChemiDoc™ XRS (Bio-Rad) apparatus. The intensity of the bands was measured by densitometry and the values obtained for the purified DDX3X were plotted as a function of the protein concentration and analysed by linear interpolation to derive a reference curve, whose slope corresponded to the estimated intensity (*I*) × ng^−1^ (DDX3X) value. This parameter was used to calculate the *I* × ng^−1^ values for the DDX3X in the cell extract, from the intensities of the DDX3X bands in the corresponding cell extract (CE) samples. The mean *I* × ng^−1^ (CE) value was used to calculate the ng of DDX3X × μl^−1^ of extract, and then the total ng of DDX3X present in the extract. This value was divided for the total number of cells used (10^7^), to derive the ng DDX3X/cell. Based on the known molecular weight of DDX3X, the Avogadro number and assuming a mean cellular volume of 6.55 × 10^−11^ l, the final molar concentration of DDX3X per cell was calculated. Each experiment was repeated three times, with each blot carrying a reference curve alongside the extract samples, to account for variations in loading/transfer efficiency. The reference I × ng^−1^ (DDX3X) value obtained was anyway comparable across the different experiments (±20% variation).

### Kinetic analysis

Time-dependent product accumulation was fitted to the simple exponential equation:

(1) $$\begin{equation*}{A}(1 - {{\rm e}^{ - kapp\,{t}}})\end{equation*}$$

where *A* is the burst constant, *k_app_* is the apparent reaction rate and *t* is time.

Dependence of the reaction products from DDX3X concentrations was fitted to the equation:

(2) $$\begin{equation*}{E_{{\rm obs}}} = {E_{{\rm max}}}/\left( {1 + {{\left( {{K_{1/2}}/{E_0}} \right)}^{n_{\rm H}}}} \right)\end{equation*}$$

where *E*_obs_ is the observed enzymatic activity in the presence of each enzyme dose *E*_0_; *E*_max_ is the maximal enzymatic activity; *n*_H_ is an exponential term to take into account sigmoidal dose–response curves.

Dose-dependent inhibition of the DDX3X nuclease reaction by ATP was fitted to the equation:

(3) $$\begin{equation*}{E_{{\rm obs}}} = {E_{{\rm max}}}/\left( {1 + {\rm{ }}{{\left( {\left[ {\rm ATP} \right]/{\rm ID_{50}}} \right)}^{n_{\rm H}}}} \right)\end{equation*}$$

where *E*_obs_ is the observed enzymatic activity in the presence of each ATP dose ([ATP]); *E*_max_ is the maximal enzymatic activity in the absence of ATP; ID_50_ is the ATP concentration inhibiting the enzymatic reaction by 50%; *n*_H_ is an exponential term to take into account sigmoidal dose–response curves.

To calculate *V*_max_ and *K*_m_ constants, data (in triplicate) were plotted and analyzed by least-squares nonlinear regression and fitted to the following equation:

(4) $$\begin{equation*} {v} = {{V}_{\rm max}}/\left( {1 + \left( {{{K}_{\rm m}}/{{\left[{\rm S} \right]}^{n}}} \right)} \right) \end{equation*}$$

where *v* is the apparent reaction velocity at each substrate concentration, *V*_max_ is the maximum rate of the reaction, *K*_m_ is the Michaelis–Menten constant, S is the variable substrate concentration, *n* is an exponential term to take into account sigmoidal dose–response curves, due to the positive cooperativity of DDX3X binding to RNA (24).

The turnover constant *k*_cat_ was calculated as *V*_max_/*(*enzyme concentration*)* and the RNA substrate binding efficiency of our enzymes was calculated as the *k*_cat_/*K*_m_ ratio.

Data were fitted to Equations (1), (2), (3) and (4) with the program GraphPad Prism 6.0.

### Transfection of HEK293T cells for engineered lentivirus preparation

For lentivirus preparation, 3–3.5 × 10^6^ HEK293T cells were seeded in 10 cm tissue culture dishes in 10 ml Dulbecco's modified Eagle's medium (Sigma) containing 10% fetal bovine serum (Sigma) and 20 μg/ml gentamycin (Sigma) and grown for 24 h in a humidified 5% CO_2_ atmosphere. After 2 h before transfection, cell medium was replaced. Then, cells were transfected with 13.2 μg Inducible Dharmacon™ TRIPZ™ Lentiviral shRNA (GE Healthcare Lafayette, United States), 10 μg pSPAX2 vector (Dharmacon™, GE Healthcare) and 4 μg pMD2 vector (Dharmacon™, GE Healthcare) diluted in 50 mM Hepes pH 7.12, 140 mM NaCl, 750 nM Na_2_HPO_4_ and 125 mM CaCl_2_. To enhance transfection efficiency, 100 μM Chloroquine (Sigma) was added to the cells. Cell medium was changed 14–16 h after transfection and viruses were collected for the two following days.

All this work was done in Biosafety Level 2 (BSL2) laboratory.

### Cells transduction and DDX3X-knockdown induction

One day prior to transduction, 500 000–600 000 U2OS cells were seeded in 2 ml Dulbecco's modified Eagle's medium (Sigma) containing 10% fetal bovine serum (Sigma) and 20 μg/ml gentamycin (Sigma) in 6 well plates and grown in a humidified 5% CO_2_ atmosphere. Then, cell medium was replaced with 2 ml of viral particles and 4 μg/ml protamine sulfate (Sigma) to enhance the transduction efficiency. After 3–4 h, new fresh medium containing viral particles was added for each plate and left O/N. Finally, cells were washed with Phosphate Saline Buffer (Sigma) and new fresh medium Dulbecco's Modified Eagle's medium (Sigma) containing 10% fetal bovine serum (Sigma) and 20 μg/ml gentamycin (Sigma) was added. Cells grown for 24 h in a humidified 5% CO_2_ atmosphere without any additional selection. Then, since cells that have integrated the Inducible Dharmacon™ TRIPZ™ Lentiviral shRNA (pTRIPZ shDDX3 130, V2THS_228965, GE Healthcare) possess Puromycin resistance, a dose-escalation curve of Puromycin (Sigma) was used to select resistant cells. All this work was done in Biosafety Level 2 (BSL2) laboratory. Knockdown (KD) of DDX3X in U2OS cells (shDDX3X cells) or in the non-silencing vector shRNA (NSC) control cells were both induced adding 1 μg/ml Doxycycline (Sigma) into the cell medium and simultaneously maintaining cells in Puromycin selection (2 μg/ml). Doxycycline was maintained for 48–72 h and then cells were harvested and KD efficiency was checked by western blot.

### siRNA-mediated protein depletion

All siRNAs were purchased from Dharmacon™-Horizon Discovery and were transfected using RNAiMAX (Invitrogen, Carlsbad, USA) according to manufacturer instructions. siRNAs were used at final concentration of 5 nM and 25 nM for DDX3X and RNaseH2 silencing, respectively. U20S cells grow in Dulbecco's modified Eagle's medium (Euroclone, Milan, Italy) containing 10% fetal bovine serum (Immunological Sciences, Rome, Italy) and 20 μg/ml gentamycin (Euroclone). Medium was changed 24 h after transfection and samples collected after 72 h. The following mRNA target sequences were used:

RNASEH2A siRNAs pool: Target sequences 5→3′
CGGGAAAGGCUGUUUGCGA; AAAUGGAGGACACGGACUU;
AUGCAUUGGACCAGGGCGU; AGACCCUAUUGGAGAGCGA
DDX3X siRNAs pool: Target sequences 5′→3′
GCAAAUACUUGGUGUUAGA; ACAUUGAGCUUACUCGUUA;
CUAUAUUCCUCCUCAUUUA; GGUAUUAGCACCAACGAGA
Non-Targeting siRNAs pool: Target sequences 5′→3′
UAGCGACUAAACACAUCAA; UAAGGCUAUGAAGAGAUAC;
AUGUAUUGGCCUGUAUUAG; AUGAACGUGAAUUGCUCAA

### Cell extracts and western blot

U20S cell pellets were lysed for 30 min on ice in 50 mM pH 8 Tris–HCl, 0.1% SDS, 350 mM NaCl, 0.25% Triton X-100 in the presence of Protease Inhibitor Cocktail 1X (Sigma). Then, samples were sonicated (at 30 s intervals) and centrifuged at 4°C for 10 min at 15 000 × g. The soluble portion of the extract was then quantified by Bradford assay (Bio-Rad) and loaded on SDS-Page gels. After gels running, proteins were transferred to a nitrocellulose membrane with a pore size of 0.45 μm (Ge Healthcare) using Trans-Blot^®^ Turbo TM device (Bio-Rad). Finally, the following primary antibodies were used: rabbit polyclonal anti-DDX3X A300-475A antibody (1:2000 dilution) (BETHYL), mouse 201055 anti-actin (1:1000 dilution) (Santa Cruz Biotechnology, Dallas, United States), mouse MAB3574 vinculin (1:10 000 dilution) (Millipore, Temecula, USA) and rabbit ab83943 anti-RNaseH2A (1:500 dilution) (Abcam, Cambridge, England).

### Riboassay

The protocol is based on the one previously published (25) with further modifications and optimization (Maffia and Sabbioneda, manuscript in preparation). Specifically, genomic DNA was extracted using NucleoSpin^®^ Tissue kit (Macherey-Nagel, Düren, Germany). Then, 1 μg of genomic DNA was digested with 1 U of *E. coli* RNaseH2 (BioLabs, Euroclone) or with 50 nM human recombinant RNaseH2 and the reaction was led in a dry heating block (Eppendorf^®^ Thermomixer^®^) at 37°C with constant shaking at 550 rpm for 2.5 h. Reaction was stopped on ice for 30 min and nick translation reaction started trough the addition of 1 U of *E. coli* DNA pol I (Biolabs, Euroclone) and 2 μl of Atto647N-dUTP pH 7.5 NT Labelling Kit (Jena Biosciences, Löbstedter, Germany) at 16°C for 1 h.

Finally, the reaction was stopped by addition of standard gel loading buffer and loaded on a 1% agarose gel at 100 V for 2 h in TBE buffer. Ethidium bromide and Atto647N-dUTP signals were detected by laser scanning densitometry (Typhoon-TRIO, GE Healthcare) and quantified with ImageQuant TL (Typhoon-TRIO, GE Healthcare) normalizing Atto647N-dUTP signals with the Ethidium bromide ones. Quantitation of undigested genomic DNAs were subtracted from quantitation of digested genomic DNAs and *P* values were calculated using the Student's *t*-test.

## RESULTS

### Human DDX3X has RNaseH2-like activity

**Figure 1.**
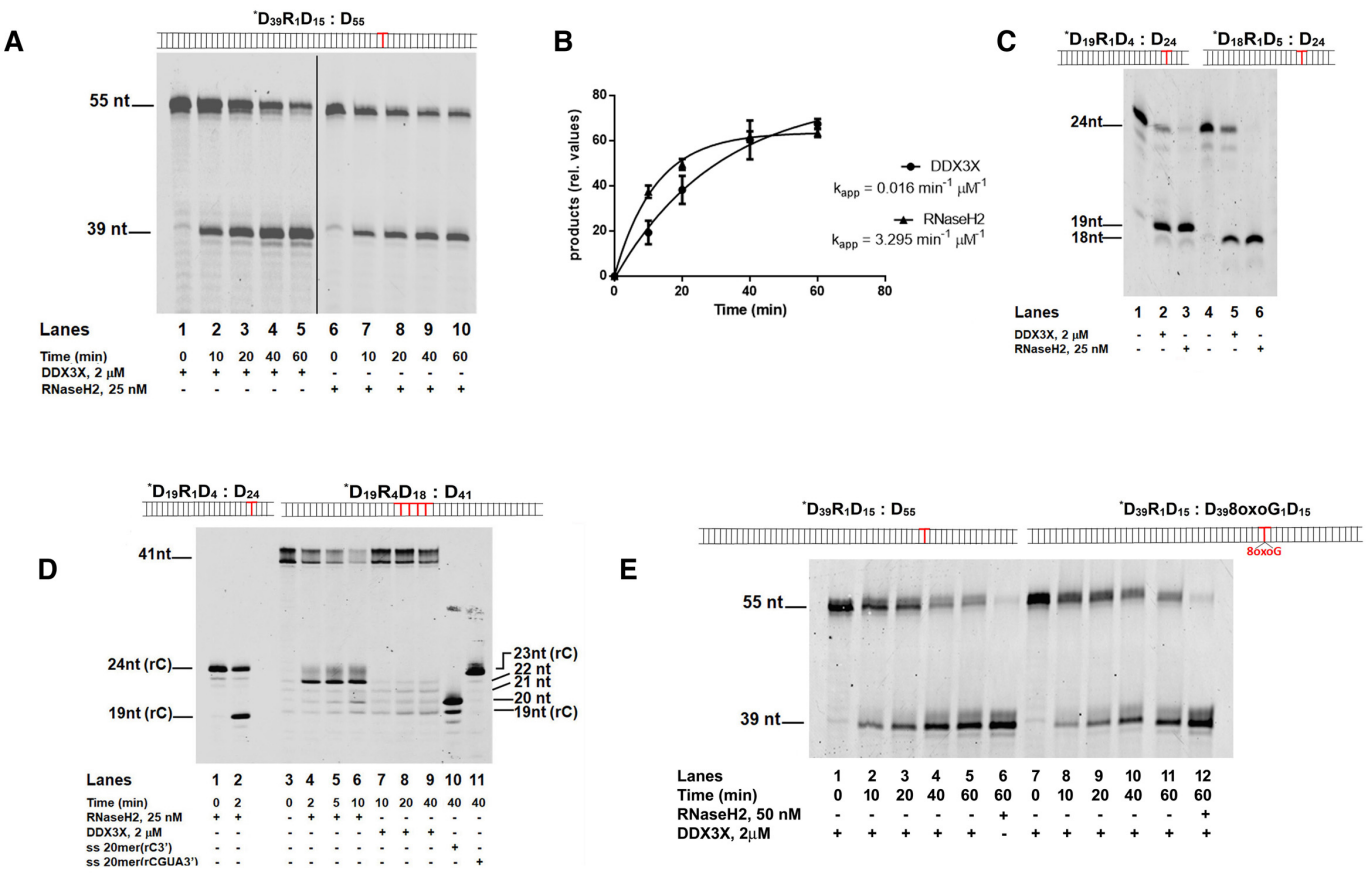
Human DDX3X has RNaseH2-like activity. (**A**) Time course of DDX3X (lanes 1–5) and RNaseH2 (lanes 6–10) digestions of Substrate *D_39_R_1_D_15_: D_55_. (**B**) Apparent digestion rates for DDX3X and RNaseH2 enzymes on Substrate *D_39_R_1_D_15_: D_55_. Values are the means of three independent estimates ± S.D. (**C**) Digestion by DDX3X (lanes 2 and 5) and RNaseH2 (lanes 3 and 6) on Substrate *D_19_R_1_D_4_: D_24_ and Substrate *D_18_R_1_D_5_: D_24_ respectively. Lanes 1 and 4: Substrates *D_19_R_1_D_4_: D_24_ and *D_18_R_1_D_5_: D_24_ alone, respectively. (**D**) Substrate *D_19_R_4_D_18_: D_41_ in the presence of RNaseH2 (lanes 4–6) or DDX3X (lanes 7–9). Lanes 1–2 control reactions with RNaseH2 and Substrate *D_19_R_1_D_4_: D_24_. Lane 3 Substrate *D_19_R_4_D_18_: D_41_ alone. Lanes 10, 11 oligonucleotide size markers of defined lengths, used to better identify the different digestion products. (**E**) Time course of DDX3X digestion of Substrate *D_39_R_1_D_15_: D_55_ (lanes 1–5) and Substrate *D_39_R_1_D_15_: D_39_8oxoG_1_D_**15**_(lanes 7–11). Control reactions with RNaseH2 of Substrate *D_39_R_1_D_15_: D_55_ (lane 6) and Substrate *D_39_**R**_1_**D**_15_**: D**_39_**8oxoG**_1_**D**_15_ (lane 12) respectively.

It is already known that DDX3X is able to resolve DNA:RNA hybrids and RNA secondary structures and it has been implicated in RNA degradation processes (14). We have been studying DDX3X protein since more than ten years, as a promising druggable antiviral and anticancer target (9). During the course of our biochemical studies, we constantly detected in our purified preparations of full length DDX3X an unexpected exoribonuclease activity of this enzyme. Being aware of preliminary implications of DDX3X in genome stability (10) and of the existence of double-functioning enzymes with helicase/RNase activities (26,27) we tested a possible DDX3X RNaseH2-like activity. When incubated in the presence of a specific oligonucleotide dsDNA substrate containing a single rCMP embedded in one strand at a specific position (Substrate *D_39_R_1_D_15_: D_55_ Figure 1A), recombinant purified human DDX3X (Supplementary Figure S1a and b) cut at the 5′ side of the single rNMP, generating a product identical to the one of RNaseH2 (Figure 1A, compare lanes 1–5 with lanes 6–10), albeit with lower efficiency (Figure 1B). Apparent rates of digestion were 0.016 min^−1^ μM^−1^ for DDX3X and 3.3 min^−1^ μM^−1^ for RNaseH2, respectively. To the best of our knowledge, no other human enzyme besides RNaseH2 has been described so far to catalyze single rNMP excision from dsDNA.

DDX3X cutting was restricted to the rNMP position and independent from the surrounding sequence, as having the rCMP at two different positions one nucleotide upstream on a different DNA substrate (Figure 1C, Substrates *D_19_R_1_D_4_: D_24_ and *D_18_R_1_D_5_: D_24_), also generated the expected products (Figure 1C, lanes 2 and 5), These products are again identical to those generated by RNaseH2 (Figure 1C, lanes 3 and 6), confirming that rCMP acted as specific recognition site for DDX3X.

On a dsDNA oligonucleotide with an embedded stretch of 4 rNMPs (Substrate *D_19_R_4_D_18_: D_41_), RNaseH2 preferentially cut 5′ of the last rNMP (28) downstream the RNA–DNA junction, generating a 22 nt product (Figure 1D, lanes 4–6). Differently, DDX3X generated 19–22 nt products (Figure 1D, lanes 7–9), with similar incision rates for each position (Supplementary Figure S1c), thus showing no preference between a DNA/RNA or an RNA/RNA phosphodiester bond.

On a dsDNA oligonucleotide with an embedded ribonucleotide opposite to an 8xoG base (Substrate *D_39_R_1_D_15_: D_39_8oxoG_1_D_15_), DDX3X cuts at the rNMP position with the same efficiency as the control substrate (Figure 1E, lanes 2–5 and Supplementary Figure S1d). This is in contrast with the reported reduced activity of RNaseH2 in removing rNMPs opposite such lesion (29).

Mass spectrometry analysis using different approaches by two independent laboratories of two different preparations of recombinant DDX3X purified from *E. coli*, showed that none of the few co-purifying *E. coli* contaminants possessed RNase or any other kind of nuclease activity (Supplementary Tables S1 and S2). The major contaminant was ferric uptake regulation protein, which binds metal ions such as the Ni^2+^ present on the medium used for affinity purification. Thus, the observed RNaseH2-like activity could be firmly attributed to DDX3X. Comparison with the related human RNA helicase DDX5 (43% identity, 60% similarity) failed to detect RNaseH2-like activity, suggesting that it was peculiar to DDX3X (Supplementary Figure S1e–g).

### RNaseH2-like and helicase activities of DDX3X are differentially regulated by ATP binding and protein oligomerization

**Figure 2.**
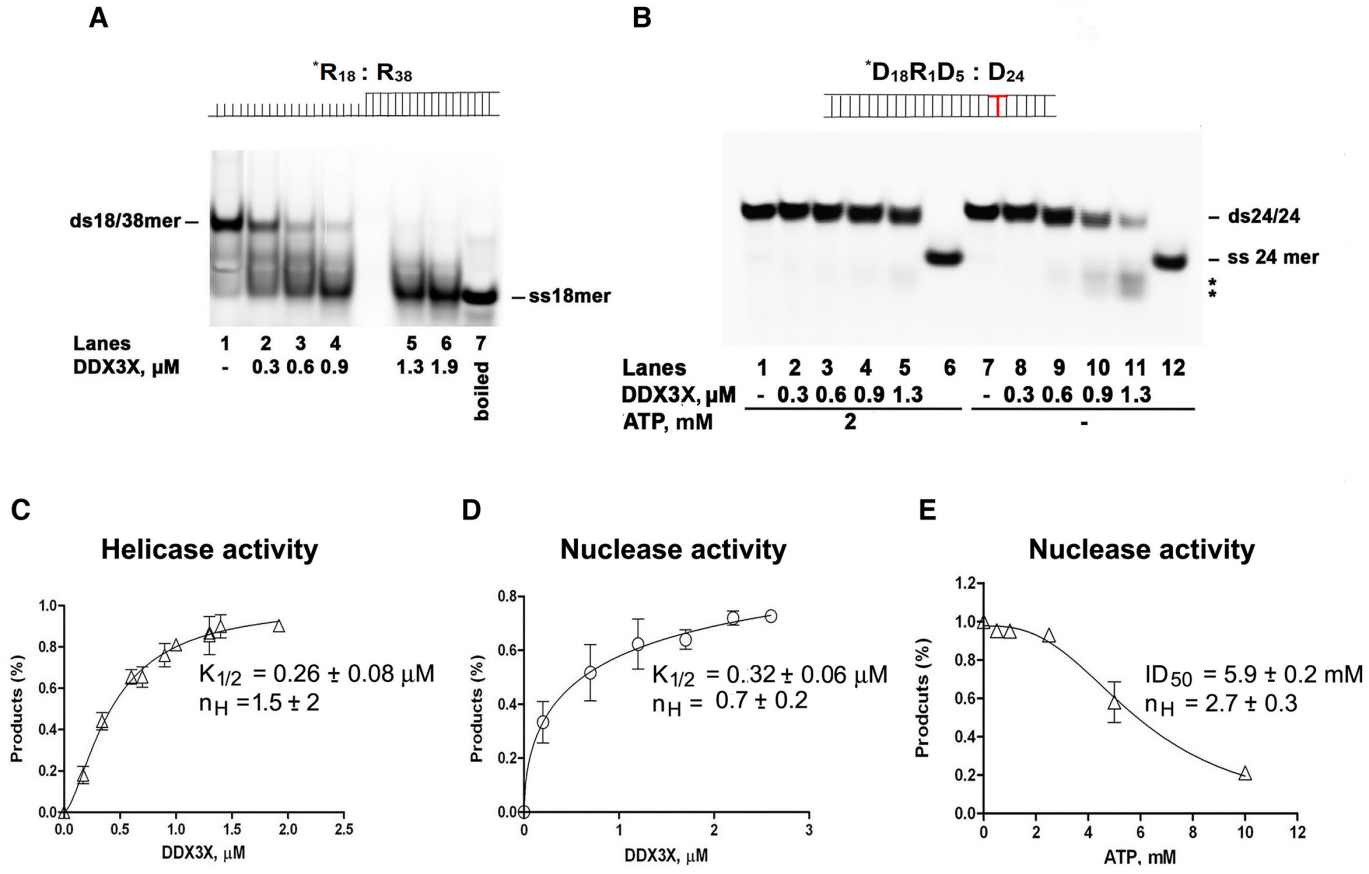
DDX3X RNaseH2-like activity is not dependent by its helicase activity and is inhibited by ATP. (**A**) Titration of DDX3X wild type (lanes 2–6) in helicase reaction with a dsRNA substrate 18/38mer with a 20 nt 3'-ss overhang (Substrate *R_18_: R_38_). Lane 1: Substrate *R_18_: R_38_ alone. Lane 7: 18mer single strand. (**B**) Native PAGE of the products of DDX3X helicase reaction with Substrate *D_18_R_1_D_5_: D_24_ in the presence (lanes 1–5) or in the absence (lanes 7–11) of ATP. Lanes 6, 12: ss24mer as marker. (**C**) Dependence of the DDX3X helicase activity from the enzyme concentration. (**D**). Dependence of the DDX3X nuclease activity from the enzyme concentration. (**E**) Inhibition of DDX3X nuclease activity by ATP. All values are the means of three independent estimates ± S.D.

In helicase assays, our DDX3X preparation preferentially unwound a dsRNA substrate with a ss-3′ overhang (Substrate *R_18_: R_38_, Figure 2A), in agreement with published results (24). As shown in Figure 2B, no unwinding was observed on the dsDNA/rNMP hybrid (Substrate *D_18_R_1_D_5_: D_24_) even at high DDX3X concentrations in the presence of ATP (Figure 2B, lanes 2–5). Without ATP (Figure 2B, lanes 8–11), at the DDX3X concentrations which cut Substrate *D_18_R_1_D_5_: D_24_ in nuclease assays, digestion products appeared (Figure 2B, lanes 10–11 marked by asterisks). Since the gel was run under non-denaturing conditions, cutting of the labelled strand at the rCMP position possibly caused dissociation of the nicked DNA strand during the gel run. Thus, the RNaseH2-like activity of DDX3X did not require its RNA helicase activity nor ATP.

In helicase assays DDX3X showed a sigmoidal dependence of unwinding with respect to the enzyme concentration (*K*_1/2_ = 0.26 μM, cooperativity index *n*_H_ = 1.5) (Figure 2C), suggesting protein oligomerization, according to published results (24). In the nuclease reaction no cooperativity was observed (*K*_1/2_ = 0.32 μM, *n*_H_ = 0.7) (Figure 2D). The nuclease activity of DDX3X was inhibited by increasing amounts of ATP (Figure 2E), with a sigmoidal dose-dependence from the ATP concentration (ID_50_ = 5.9 mM, *n*_H_ = 2.7), suggesting that binding of ATP triggered DDX3X oligomer formation, as suggested for other DEAD-box helicases (30). Thus, DDX3X apparently interacted with the nucleic acid substrate with a different stoichiometry when acting as a nuclease rather than a helicase and its oligomeric state was regulated by ATP (31,32).

### Sequence and structural comparison reveal conserved amino acids in the catalytic site of RNaseH2 and DDX3X

**Figure 3.**
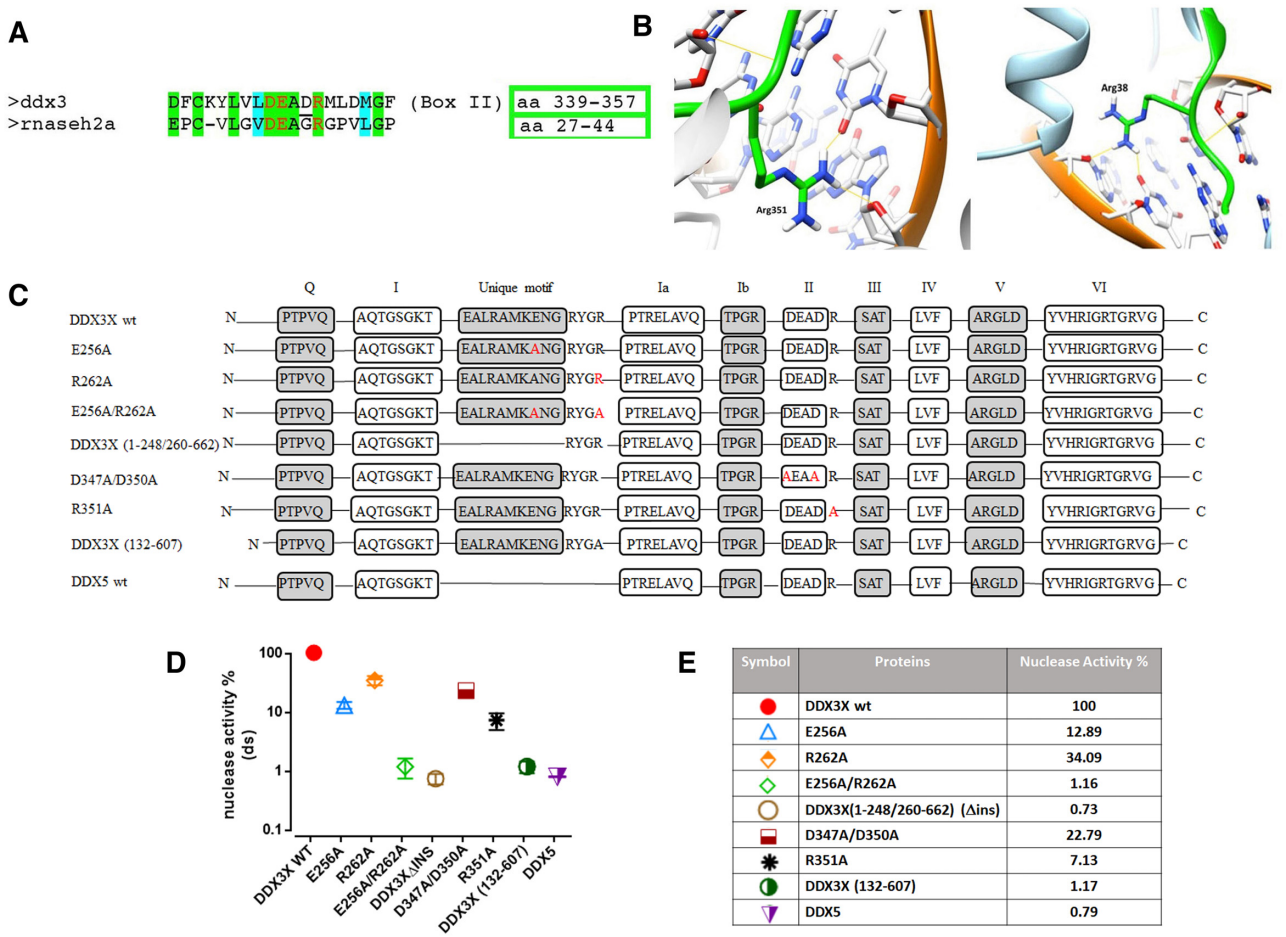
Identification of DDX3X residues important for its RNaseH2-like activity. (**A**) Sequence alignment showing amino acid (aa) similarities between the conserved -DEAD- (box II) helicase motif of DDX3X and the catalytic site of human RNaseH2. Red letters indicate catalytically important residues. Green shading indicated identical and cyan shading similar aa. The numbers of the aa residues corresponding to each selected region are boxed. (**B**) Structure comparison between human DDX3X in open conformation (PDB ID: 5e7I) on the left and mouse RNaseH2 (PDB ID: 3KIO) of the region surrounding Arg351 of DDX3X (left) and Arg38 of RNaseH2 (right), showing the hydrogen bond formed by these residues with the RNA substrate. Arg351 in DDX3X and Arg38 in RNase H2 were similarly positioned within these regions. (**C**) Graphical representation of all the DDX3X mutants and DDX5 protein. N and C indicate the N-terminal and C-terminal of the proteins respectively. Boxes represent all the conserved motifs of DEAD-box proteins (with the addition of DDX3 unique motif). Red letters indicate single aa mutations. (**D**) Nuclease activities of the DDX3X mutants and DDX5 protein relative to DDX3X wild type (taken as 100%). All catalytic activities were assessed on Substrate *D_39_R_1_D_15_: D_55_. Values are the mean of four independent experiments ± S.D. All proteins were tested at 2 μM. (**E**) Summary table of the catalytic activities shown in panel D.

Attempting to rationalize the observed nuclease activity, previously unnoticed in human DDX3X or its orthologs in other organisms, a structural modeling comparison was made between catalytically important regions of RNaseH2 and DDX3X. Because of the lack of complete crystal structures of human RNaseH2, we selected the structure of the murine enzyme (amino acid identity 86.62%). The DDX3X crystal structure in its open conformation was used (33) since our results indicated that DDX3X acts as a monomer (i.e. in open conformation) in its RNaseH2-like activity. Sequence (Figure 3A) and structural (Figure 3B) alignments revealed that Arg38 of the RNaseH2 catalytic motif -DEAGR- (28) could be aligned to Arg351 of the DDX3X catalytic motif -DEADR- (34) both residues taking similar interactions with the RNA substrate in both enzymes (Figure 3B). Such interactions were lost in the recently published structure of DDX3X in a closed dimeric conformation bound to dsRNA (31) supporting our hypothesis of a monomeric conformation of DDX3X when displaying its RNaseH2-like activity.

### The RNaseH2-like activity of DDX3X requires its RNA and ATP binding domains

To verify our modeling, we mutated Arg351 into Ala. The purified R351A DDX3X mutant (Supplementary Figure S2a), displayed a *k*_cat_/*K*_m_ reduction of 5.5-fold in helicase activity if compared to wild type (Supplementary Figure S2b). More interestingly, R351A displayed also >90% reduced nuclease activity with respect to the wild type (Figure 3D, E), similarly to the reported strongly reduced nuclease activity of RNaseH2 mutant R38A (35). To further identify other domains/residues responsible for the nuclease activity of DDX3X, we used a series of DDX3X mutants (Figure 3C and Supplementary Figure S2a) available in our laboratory.

The DDX3X double mutant D347A/D350A (DADA) lacking ATPase/helicase activity (36) also displayed reduced nuclease activity (Figure 3D and E). A deletion mutant (1–248/260–662) lacking the DDX3X-specific 10 aa insertion (aa 249–259) named ‘unique motif’, important for RNA recognition, was shown to have reduced nucleic acid binding and helicase activity (36). It showed almost undetectable nuclease activity with respect to wild type (Figure 3D and E). Ongoing molecular modeling and biochemical studies in our laboratory identified Glu256 inside this motif and Arg262 four residues immediately downstream, as important for RNA interaction and unwinding (37). The mutants E256A and R262A also showed strongly reduced RNaseH2-like activity (Figure 3D and E) and the double mutation E256A/R262A almost completely abolished DDX3X nuclease activity (Figure 3D and E). Next, we observed that the minimal catalytic core of DDX3X (aa 132–607) showed no detectable nuclease activity (Figure 3D and E), despite conserved helicase and ATPase activities (15). Interestingly, the closely related enzyme DDX5 also did not show nuclease activity despite the presence of conserved ATPase and RNA binding domains. DDX5 bears different N- and C-terminal domains with respect to DDX3X and it lacks the DDX3X unique motif. Thus, the observed RNaseH2-like activity of DDX3X requires its ATPase and RNA binding domains, including the unique motif in addition to the N- and C-terminal domains which are not present in the minimal catalytic core (aa 132–607).

### *In vitro* reconstitution of ribonucleotide excision repair with specialized DNA polymerases β and λ

**Figure 4.**
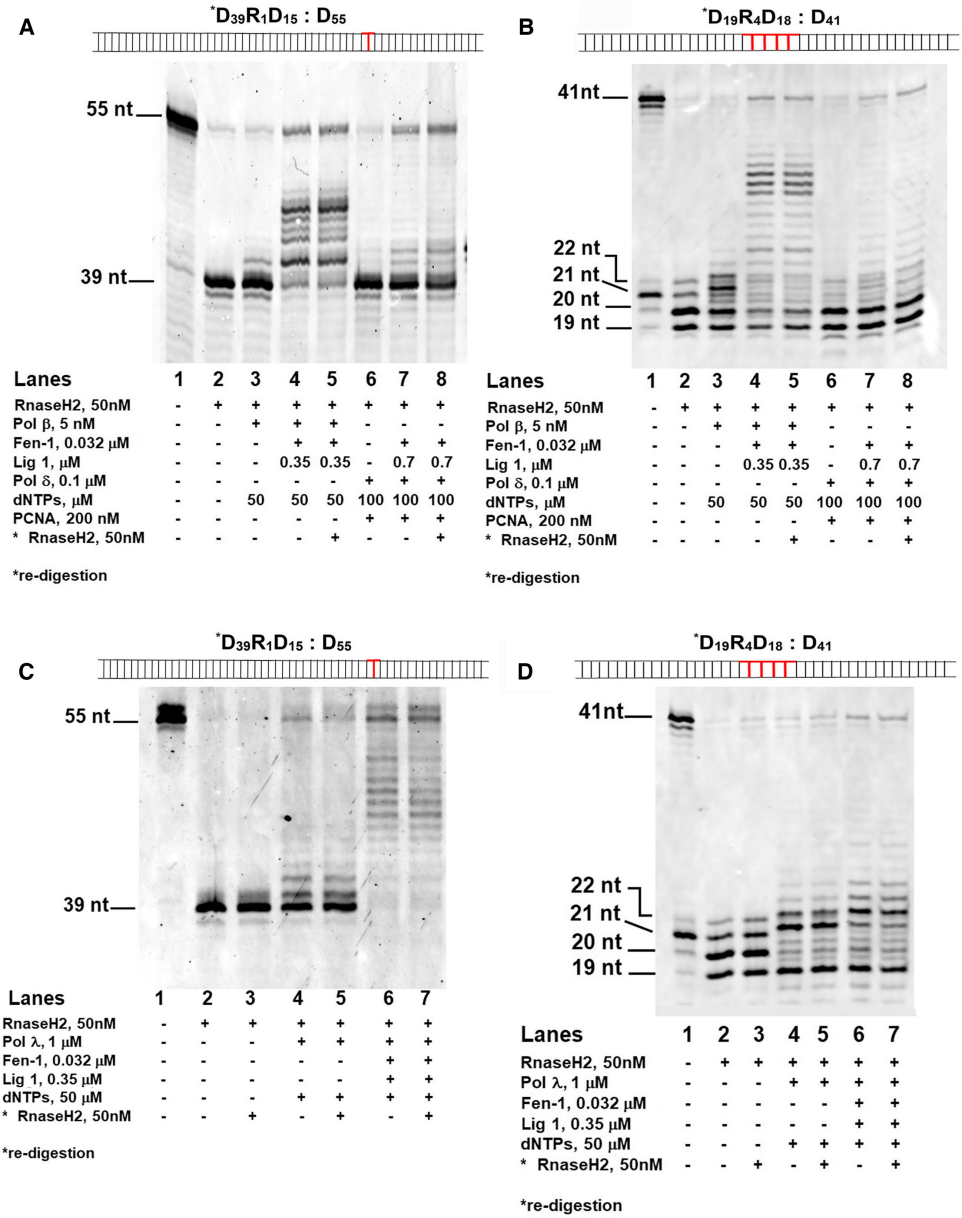
Different DNA polymerases support full ribonucleotide excision repair together with RNaseH2. (**A**) RER reactions in the presence of RNaseH2, Pol β and dNTPs alone (lane 3), or with DNA ligase 1 and Fen-1 (lanes 4–5). Lane 5: re-digestion by RNaseH2 of the ligated products after heat inactivation of the reaction. RER reactions in the presence of RNaseH2, Pol δ, PCNA and dNTPs (lane 6), or with added DNA ligase 1 and Fen-1 (lanes 7–8). Lane 8: re-digestion by RNaseH2 as in Lane 5. Lane 1: Substrate *D_39_R_1_D_15_: D_55_ alone. (**B**) RER reactions in the presence of RNaseH2, Pol β, dNTPs, Fen-1 and DNA ligase 1 (lanes 2–5). RER reactions in the presence of RNaseH2, Pol δ, PCNA and dNTPs (lanes 6–8), DNA ligase 1 and Fen-1 (lanes 7–8). Lanes 5 and 8: re-digestion by RNaseH2. Lane 1: Substrate *D_19_R_4_D_18_: D_41_ alone. (**C**) RER reactions in the presence of RNaseH2 (lanes 2–3), Pol λ, dNTPs (lanes 4–5), Fen-1 and DNA ligase 1 (lanes 6–7). Lanes 3, 5 and 7: re-digestion by RNaseH2. Lane 1: Substrate *D_39_R_1_D_15_: D_55_ alone. (**D**) Lane 1: Substrate *D_19_R_4_D_18_: D_41_ alone. RER reactions in the presence of RNaseH2 (lanes 2–3), Pol λ, dNTPs (lanes 4–5), Fen-1 and DNA ligase 1 (lanes 6–7). Lanes 3, 5 and 7: re-digestion by RNaseH2.

It is still unclear whether other Pols, besides Pols δ/ϵ, might perform the synthesis step of RER pathway in eukaryotes. In order to reconstitute the full RER reaction *in vitro*, we incubated human RNaseH2, human Fen-1 and human DNA ligase 1 not only with the canonical human Pol δ, but also in combination with the X-family DNA repair enzymes human Pols β and λ on a dsDNA substrate bearing a single rCMP at position +39 (Substrate *D_39_R_1_D_15_: D_55_) and a dsDNA oligonucleotide with an embedded stretch of four rNMPs (Substrate *D_19_R_4_D_18_: D_41_) both long enough to allow productive binding by Fen-1. Starting from Substrate *D_39_R_1_D_15_: D_55_, RNaseH2 generated the expected 39 nt product (Figure 4A, lane 2), allowing subsequent incorporation by Pol β in the presence of dNTPs at their intracellular physiological range (50 μM) (Figure 4A, lane 3). Addition of Fen-1 and DNA ligase 1 allowed flap removal and ligation, generating a fully ligated 55 nt repair product (Figure 4A, compare lane 3 with lane 4). The flap cutting activity of our Fen-1 was independently checked and confirmed on a canonical flap substrate (Supplementary Figure S2c). This product was resistant to degradation by RNaseH2 added back after the ligation step, indicating that it did not contain rCMP (Figure 4A, lane 5). In an equivalent experiment with Pol δ and PCNA (using 100 μM dNTPs), strand displacement synthesis (Figure 4A, lane 6) was followed by formation of RNaseH2-resistant ligated 55 nt product only in the presence of both Fen-1 and DNA ligase 1 (Figure 4A, compare lane 7 with 8). In the presence of a stretch of 4 rNMPs (Substrate *D_19_R_4_D_18_: D_41_) and RNaseH2, reactions with Pol β and δ in the conditions previously described, generated a fully ligated 41 nt repair product only in presence of Fen1 and DNA ligase 1 (Figure 4B, compare lane 3 with 4 for Pol β activity and lane 6 with 7 for Pol δ). These products were resistant to degradation by RNaseH2 added back after the ligation steps, indicating that they did not contain rNMPs and that fully repair products could be obtained also starting from a stretch of four rNMPs with both canonical and non-canonical polymerases (Figure 4B, lane 5 for Pol β and lane 8 for Pol δ).

We observed that also the DNA repair Pol λ could operate in RER. Performing assays with both Substrates *D_39_R_1_D_15_: D_55_ and *D_19_R_4_D_18_: D_41_, in the presence of dNTPs at their intracellular physiological range (50 μM), RNaseH2 generated the expected products (Figure 4C, lane 2 and Figure 4D, lane 2 respectively), addition of Pol λ resulted in nucleotide incorporation (Figure 4C, lane 4 and Figure 4D, lane 4 respectively) and adding both Fen-1 and DNA ligase 1, fully ligated repair products appeared (Figure 4C, compare lane 2 with lane 4 for Substrate *D_39_R_1_D_15_: D_55_; Figure 4D, compare lane 2 with lane 4 for Substrate *D_19_R_4_D_18_: D_41_). These products were resistant to degradation by RNaseH2 added back after the ligation steps, indicating that they did not contain rNMPs (Figure 4C, lane 7 for Substrate *D_39_R_1_D_15_: D_55_ and Figure 4D, lane 7 for Substrate *D_19_R_4_D_18_: D_41_).

### Specialized DNA polymerases support ribonucleotide excision repair also with DDX3X

**Figure 5.**
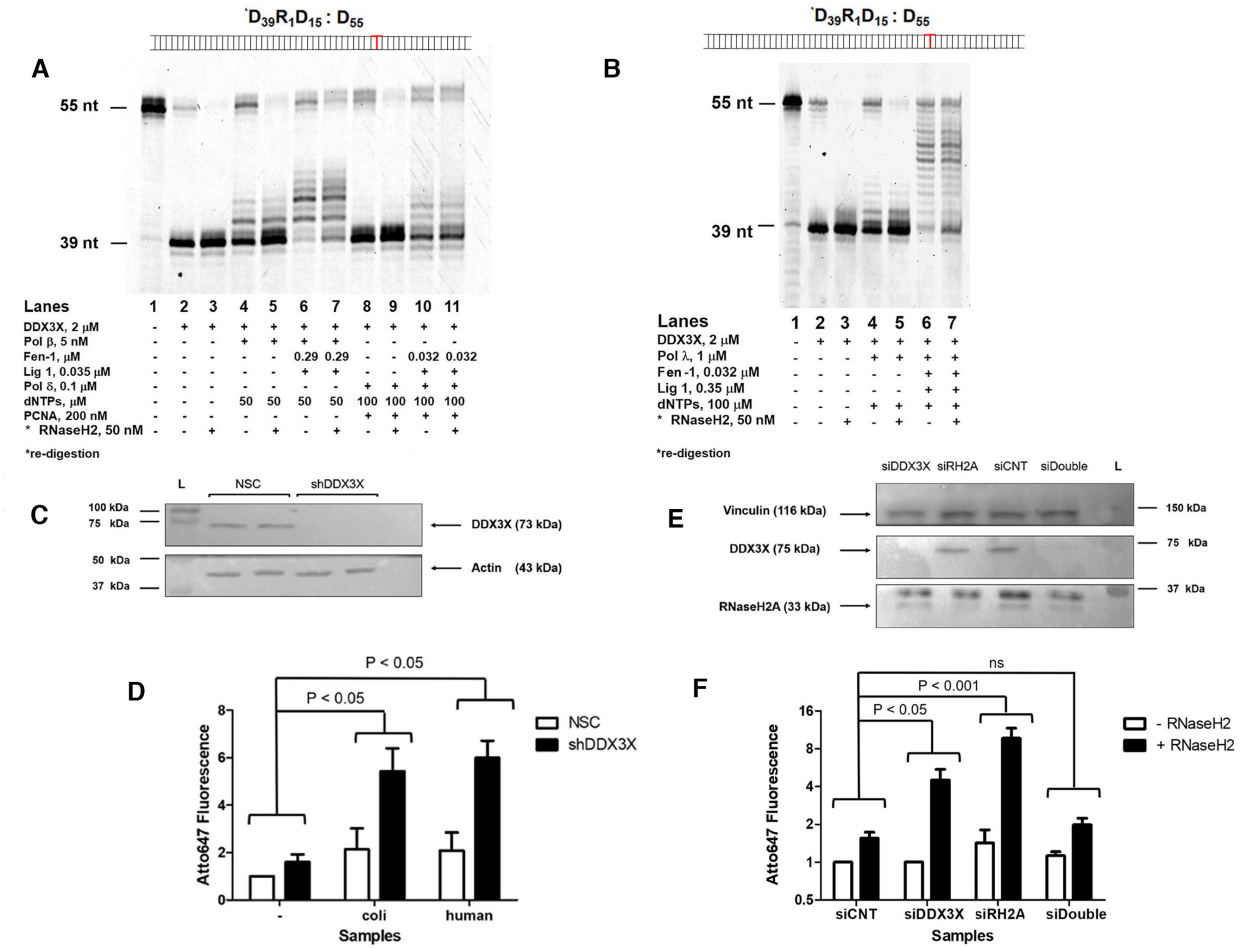
A role of DDX3X in ribonucleotide excision repair. (**A**) RER reactions in the presence of DDX3X (lanes 2–3), Pol β, dNTPs (lanes 4–5), Fen-1 and DNA ligase 1 (lanes 6–7). Lanes 3, 5, 7: re-digestion by RNaseH2 of the heat inactivated reactions. RER reactions in the presence of DDX3X, Pol δ, PCNA, dNTPs (lanes 8–9), Fen-1 and DNA ligase 1 (lanes 10–11). Lanes 9 and 11: re-digestion by RNaseH2. Lane 1: Substrate *D_39_R_1_D_15_: D_55_ alone. (**B**) RER reactions in the presence of DDX3X (lanes 2–3), Pol λ, dNTPs (lanes 4–5), Fen-1 and DNA ligase 1 (lanes 6–7). Lanes 3, 5 and 7: re-digestion by RNaseH2. Lane 1: Substrate *D_39_R_1_D_15_: D_55_ alone. (**C**) Western blot for DDX3X and β-actin. L, molecular weight markers. NSC, non-silencing vector control cells; shDDX3X, stable DDX3X-silenced cells. (**D**) Quantification of genomic rNMPs by the riboassay. NSC untreated cells signal was set as 1 fluorescence arbitrary unit. Different treatments were: untreated genomic DNA (–), genomic DNA treated with 1 U of *E. coli* RNaseH2 or with 50 nM of human recombinant RNaseH2 (+). Data represent mean ± SEM of at least four independent experiments. (**E**) Western blot for vinculin, DDX3X and RNaseH2A. L, molecular weight markers. siCNT, non-silencing smart-pool siRNAs; siDDX3X, DDX3X-silenced cells, siRH2A, RNaseH2A-silenced cells. (**F**) Quantification of genomic rNMPs by the riboassay. siCNT (control) cells signal was set as 1 fluorescence arbitrary unit. As above, different treatments were: untreated genomic DNA (–) and genomic DNA treated with 50 nM of human recombinant RNaseH2 (+). Data represent mean ± SEM of at least four independent experiments.

Under similar conditions, we repeated the experiments substituting RNaseH2 with DDX3X. Starting from Substrate *D_39_R_1_D_15_: D_55_ and using Pol β and Pol δ under the same conditions employed for RNaseH2, also with DDX3X a 55 nt repair product was generated in the presence of Fen-1 and DNA ligase 1 (Figure 5A, lane 6 for Pol β and lane 10 for Pol δ), which was resistant to degradation by RNaseH2 added back after the ligation step (Figure 5A, lane 7 for Pol β and lane 11 for Pol δ). Also using Pol λ, we were able to obtain a 55 nt repair product in the presence of Fen-1 and DNA ligase 1 resistant to degradation by RNaseH2 added back after the ligation step (Figure 5B, compare lane 6 with lane 7).

Thus, our *in vitro* results indicated that both DDX3X and RNaseH2 can initiate RER in cooperation with different Pols.

### Non-overlapping roles of DDX3X and RNaseH2 in ribonucleotide excision repair

To assess whether DDX3X acts as an alternative RNaseH2-like enzyme in cells, even in an RNaseH2 wild type genetic background, we quantified the amount of genomic rNMPs by a specific riboassay (see Materials and Methods), in cells silenced for DDX3X. If our hypothesis is true, we expected to see an accumulation of rNMPs in silenced cells compared to the control. Stable DDX3X knockdown (KD) U2OS cells were generated by lentiviral transduction of a specific shRNA and silencing efficiency was assessed by Western Blot (Figure 5C).

We found that the level of genomic rNMPs in stable DDX3X KD cells was 3-fold higher than in the non-silencing vector control (NSC) cell line after both *E. coli* RNaseH2 and human RNaseH2 treatments with a *P* value significance < 0.05 (Student's *t*-test) (Figure 5D, compare the fluorescence increase between NSC untreated and *E. coli* RNaseH2- treated cells and shDDX3 untreated and *E. coli* RNaseH2- treated cells). This result demonstrated that DDX3X KD U2OS cells are more prone to accumulate rNMPs in their genome compared to not silenced cells, even in the presence of a functional RNaseH2, suggesting that in cells, DDX3X and RNaseH2 operate in alternative, non-redundant RER pathways. To complement this approach we then silenced DDX3X and RNaseH2, alone or in combination, also using siRNA technology. Silencing efficiency was assessed by Western Blot (Figure 5E) and again we show that DDX3X KD cells accumulate 3-times more ribonucleotides compared to control in accordance with our previous findings (Figure 5F). RNaseH2 silencing causes an abundant ribonucleotides accumulation (Figure 5F) as previously published (25) while the silencing of both enzymes did not result in an additive effect (Figure 5F). This result could be either explained by the lower efficiency of silencing in the double knockdowns samples or by the activation of an alternative pathway since both RNaseH2 and DDX3X were depleted (7,8).

## DISCUSSION

According to the current models, error-free removal of rNMPs by RER absolutely relies on one key enzyme: the ribonuclease RNaseH2, the only enzyme known so far to be able to recognize and incise the DNA-RNA junction at the 5′ side of a single rNMP embedded into a double stranded DNA. This elegant model, however, raises additional questions. For example, it has been shown so far that in the absence of RNaseH2, DNA topoisomerases can initiate and aberrant, highly mutagenic rNMP removal reaction, leading to DNA deletions. Thus, it could be relevant for the cell to have other less mutagenic, backup alternative mechanisms for rNMPs removal, which leads to the question as to whether other enzymes besides RNaseH2 could initiate RER. In addition, experimental evidence, also provided by our group, suggest that repair Pols can incorporate rNMPs (16,29). This raises the problem of which Pol might operate in cleaning the rNMPs incorporated as a consequence of DNA repair, in RER of post-mitotic cells, such as neurons for example, where replicative Pols δ and ϵ are not expressed.

In the present manuscript, we offer a possible answer to both these questions, by disclosing two absolutely novel, to the best of our knowledge, findings: the existence of a second RNaseH2-like enzyme in human cells, the RNA helicase DDX3X, and the ability of repair Pols β and λ to allow full RER reactions.

In our *in vitro* systems, DDX3X operated with an apparent lower cutting efficiency than RNaseH2 (Figure 1B). However, it should be noted that the calculated values only represent a lower limit estimate of efficiency, assuming that all DDX3X molecules present in the reaction were active and the preparation homogeneous, which was only approximately correct (see mass spectrometry results, Supplementary Tables S1 and S2). More importantly, DDX3X is known to act as a scaffold for multiprotein complexes (9) thus it is possible that auxiliary factors or post-translational modifications might also modulate *in vivo* its efficiency in this context.

Mass spectrometry analysis, combined with molecular modeling-guided site-directed mutagenesis, confirmed that the observed RNaseH2-like activity could be firmly attributed to DDX3X. The closely related human RNA helicase DDX5 (43% identity, 60% similarity) failed to show RNaseH2-like activity, also suggesting that it was peculiar to DDX3X. In addition, our kinetic data show that the RNaseH2-like activity of DDX3X did not require its RNA helicase activity nor ATP. Moreover, they suggest that the helicase vs. nuclease activities of DDX3X are regulated by ATP, through the induction of different oligomeric states, thus providing a possible mechanism for the modulation of these two activities into the cell. Such a role of ATP as a regulator of the stoichiometry of the interaction of DDX3X and RNA is in agreement with the findings of Kim and Myong (32). The current model of helicase activity for DDX3X implies that the enzyme acts as a multimer (trimer) whose formation is triggered by ATP binding and that ATP hydrolysis results its dissociation. Kim and Myong showed that DDX3X undergoes a concentration-dependent oligomerization, and ATP negatively impacted on the affinity of the multimeric form for RNA, promoting its dissociation. In monomeric form, the enzyme interacts differently with an ssRNA molecule inducing a tight compaction of the nucleic acid. Such a conformation, where the protein tightly wraps around the nucleic acid strand tightly, would be the most favorable also for sensing and processing a single rNMP within a DNA strand. Our data show that DDX3X, differently from RNaseH2, is very efficient in cutting a single rNMP embedded within a ssDNA strand (Supplementary Figure S1h), consistent with this model. Thus, ATP by inducing a different stoichiometry/affinity for the substrate, shifts the equilibrium from a nuclease-competent to a helicase-competent form.

The possibility shown here, to fully reconstitute RER reactions *in vitro* by various combinations of RNaseH2 and DDX3X with at least three different Pols (Pol δ, pol β and pol λ), along with the observation that silencing DDX3X causes rNMPs accumulation in the genome, even in a RNaseH2 wild type background, suggests that RNaseH2 and DDX3X operate in a non-redundant fashion. Probably, in cells the local availability of a particular Pol along with either RNaseH2 or DDX3X, can be exploited by the different alternative RER pathways. We are nonetheless well aware of the fact that our *in vitro* system represents an oversimplification of the *in vivo* situation, which need to be validated in more physiological systems.

However, the potential relevance of our findings stretches from showing the possibility of an efficient mechanism of RER in non-replicating cells, to the suggestion that RNaseH2-deficient cells might still, at least in part, counteract rNMPs accumulation, but less efficiently. Indeed, accumulation of rNMPs in the genome above a certain threshold, induces cellular stress also in the presence of a completely functional RNaseH2 (4). DDX3X seems crucial during embryogenesis (13) while in adult cells some of its functions could be compensated by other DEAD-box proteins (38). However, loss of DDX3X correlates with increased DNA damage accumulation (13). DDX3X (11) and Pols β and λ (39) are often overexpressed in solid tumors. Our quantification of the DDX3X protein concentrations in several tumor cell lines, revealed that many of them have levels of DDX3X close to the concentrations used in our *in vitro* systems (Supplementary Table S3). Thus, alternative RER-pathways might contribute to the tolerance of high levels of rNMPs in the genome by tumor cells.

As already mentioned, loss-of-function mutations of RNaseH2 cause the neurodegenerative disease Aicardi-Goutieres Syndrome (28) through activation of innate immunity (40). DDX3X promotes innate immune responses (41) and hypomorphic mutations of DDX3X have been linked to neurological disorder and mental retardation (12). Thus, our findings of its RNaseH2-like activity, may suggest a role of this protein also in the pathogenesis of AGS, through the differential processing of a specific subset of DNA:RNA hybrids. In this context, it would be interesting to assess the level of expression of DDX3X in cells from AGS patients, as well as to evaluate their sensitivity towards DDX3X silencing.

Overall, the results presented here challenge the current RER model, significantly broadening the landscape of possible alternative mechanisms that can be used by human cells to cope with the burden of misincorporated rNMPs.

## DATA AVAILABILITY

All the original raw data are available from the authors upon reasonable request.

## Supplementary Material

gkaa948_Supplemental_File

## Notes

Present address: Federica Casiraghi, CIBIO, Department of Cellular, Computational and Integrative Biology University of Trento, Via Sommarive 9, I-38123 Povo (TN), Italy.

Present address: Antonio Maffia, University of California Berkeley, Department of Molecular and Cell Biology, Berkeley, CA 94720, USA.
