# Correction to ‘Novel alternative ribonucleotide excision repair pathways in human cells by DDX3X and specialized DNA polymerases’

**DOI:** 10.1093/nar/gkaf578

**Published:** 2025-06-23

This is a correction to: Valentina Riva, Anna Garbelli, Federica Casiraghi, Francesca Arena, Claudia Immacolata Trivisani, Assunta Gagliardi, Luca Bini, Martina Schroeder, Antonio Maffia, Simone Sabbioneda, Giovanni Maga, Novel alternative ribonucleotide excision repair pathways in human cells by DDX3X and specialized DNA polymerases, Nucleic Acids Research, Volume 48, Issue 20, 18 November 2020, Pages 11551–11 565, https://doi.org/10.1093/nar/gkaa948

In February 2025, the Editors were made aware of a potential splice line between the siCNT and siDouble lanes in Fig. 5E.

The authors have provided the following explanation:

“The purpose of panel E was to demonstrate that silencing DDX3X and RNaseH2, either individually or together, was effective in our experiments measuring ribonucleotide incorporation into the genome. Figure 5E was assembled during the paper's revision to help determine the optimal final figure that would best summarize all relevant information. This panel was a temporary placeholder for internal use only and contained two spliced bands as references. It was intended to be replaced with the experimental figure before submission to the journal. Unfortunately, due to an oversight, the placeholder image was mistakenly uploaded instead of the final corrected version shown below. While we acknowledge and apologize for this mistake, we believe it does not affect the validity of the findings presented in the manuscript.”


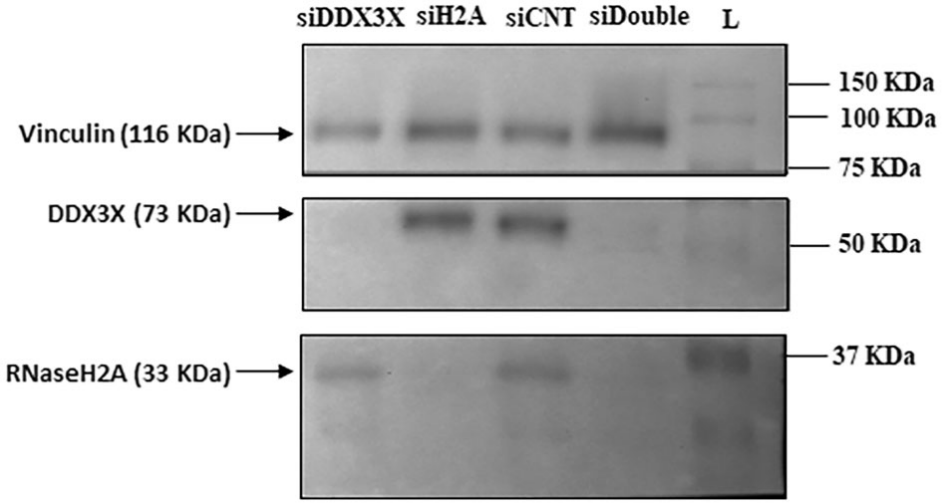


**New Figure 5E**

Copies of the lab books with the original data are in the supplementary data accompanying this correction notice.

This correction does not affect the results, discussion and conclusions presented in the article. Figure 5E has been corrected only in this correction notice to preserve the published version of record.

## Supplementary data

Supplementary data is available at NAR online.

## Supplementary Material

gkaf578_Supplemental_Files

