# Supplementary material for: Novel alternative ribonucleotide excision repair pathways in human cells by DDX3X and specialized DNA polymerases
Source: Nucleic Acids Res. 2020 Nov 2;48(20):11551–65. doi: 10.1093/nar/gkaa948 (PMC7672437; doi:10.1093/nar/gkaa948)
Supplement: gkaa948_Supplemental_File [file gkaa948_supplemental_file.pdf]

Supplementary Information to the manuscript:

Novel alternative ribonucleotide excision repair pathways in human cells by DDX3X and specialized DNA polymerases

Valentina Riva[1†], Anna Garbelli[1†], Federica Casiraghi[1‡], Francesca Arena[1], Claudia Trivisani[2], Assunta Gagliardi[3], Luca Bini[3], Martina Schroeder[4], Antonio Maffia[1+], Simone Sabbioneda[1], and Giovanni Maga[1*]

1. Institute of Molecular Genetics IGM-CNR "Luigi Luca Cavalli-Sforza", via Abbiategrasso 207, I-27100 Pavia (Italy). 2. Department of Biotechnology, Chemistry and Pharmacy, University of Siena, Via A. De Gasperi 2, I-53100 Siena (Italy). 3. Department of Life Sciences, Via A. Moro2, University of Siena, I-53100 Siena (Italy). 4. Kathleen Lonsdale Institute for Human Health Research, Biology Department, Maynooth University, Maynooth, Co. Kildare (Ireland).

† These authors equally contributed to this work

‡ Current address: CIBIO, Department of Cellular, Computational and Integrative Biology University of Trento, Via Sommarive 9, 38123 Povo (TN) Italy

+ Current address: UC Berkeley, Department of Molecular and Cell Biology, Berkeley, CA 94720, USA

Contents:

Supplementary Tables S1-3

Supplementary Figure Legends

Supplementary Figures S 1-2

Supplementary Table S1: Identified proteins from Liquid chromatography–tandem MS (LC–MS/MS) analysis

#	Identified Proteins	UniProt ID	Molecular Weight	Host	Exclusive Unique Peptide Count
1	ATP-dependent RNA helicase DDX3X	O00571	73 kDa	*Homo sapiens*	25
2	Ferric uptake regulation protein	P0A9A9	17 kDa	*E. coli* (Strain K12)	7
3	Chaperone protein DnaK	P0A6Y8	69 kDa	*E. coli* (Strain K12)	6
4	60 kDa chaperonin	P0A6F5	57 kDa	*E. coli*	5
5	Bifunctional polymyxin resistance protein ArnA	P77398	74 kDa	*E. coli* (Strain K12)	4
6	Pyruvate kinase II	P21599	51 kDa	*E. coli* (Strain K12)	4
7	cAMP-activated global transcriptional regulator CRP	P0ACJ8	24 kDa	*E. coli* (Strain K12)	3
8	Acyl-[acyl-carrier-protein]-UDP-N-acetylglucosamine O-acyltransferase	P0A722	28 kDa	*E. coli*	3
9	Protein RecA	P0A7G6	38 kDa	*E. coli*	3

				(Strain K12)	

Supplementary Table S2: Identified proteins from 2D gel by MALDI-TOF/TOF MS

#	Identified Proteins	UniProt ID	p*I*/MW (kDa)[a]	Host	Mascot search results[b]		
					Score	N. of peptides	Sequence coverage (%)
a	ATP-dependent RNA helicase DDX3X	O00571	6.17/79.8	*Homo sapiens*	104	10/14	13
b	ATP-dependent RNA helicase DDX3X	O00571	6.43/79.8	*Homo sapiens*	354	35/52	48
c	ATP-dependent RNA helicase DDX3X	O00571	6.28/79.8	*Homo sapiens*	208	18/21	23
d	60 kDa chaperonin	Q0T9P8	4.85/56.7	*E. coli*	118	8/8	17
e	Elongation factor Tu 1	A7ZSL4	5.42/44.9	*E. coli*	167	13/21	31
f	Elongation factor Tu 1	A7ZSL4	5.32/44.7	*E. coli*	111	8/12	22
g	Elongation factor Tu 1	A7ZSL4	5.38/45.4	*E. coli*	73	5/7	12
h	Elongation factor Tu 1	A7ZSL4	5.40/45.0	*E. coli*	116	8/10	19
i	Ferric uptake regulation protein	P0A9B1	5.73/17.4	*E. coli*	85	5/5	33
j	Ferric uptake regulation protein	P0A9B1	5.80/17.3	*E. coli*	189	11/15	71
k	Purine nucleoside phosphoramidase	P0ACE8	5.69/11.5	*E. coli*	197	11/15	68

[a]p*I*/MW= Experimental Isoelectric point/Experimental Molecular Weight

[b]MASCOT search results with score corresponding to MASCOT score (Matrix Science, London,UK; http://www.matrixscience.com),number of matched peptides/number of searched peptides, sequence coverage % (number of the identified residues/total number of amino acid residues in the protein sequence).

Supplementary Table S3: Estimated intracellular DDX3X protein concentrations in different cell lines

**Cell line**	**DDX3X (nM)[a]**
HeLa	124±20
U2OS	174±15
DU145	372±20
VEROE6	421±20
HUH-7	845±20
HepG2	539±50
HN6	564±40

a. DDX3X concentrations were calculated as described in the Methods section.

Supplementary Figure S1. Purification of recombinant wild type human DDX3X and DDX5 and assessment of nuclease activity of DDX5. a. Coomassie blue stained SDS-PAGE of human recombinant full length DDX3X expressed in *E. coli* and purified from a Ni-NTA column. Solid line indicates two portions of the same gel brought next for clarity. **b.** Western blot of human recombinant full length DDX3X of the same fraction shown in panel a. Recombinant DDX3X is detected using an anti-DDX3X polyclonal antibody A300-474A (BETHYL). **c.** Apparent rate constants (k_{app}) for digestion of DDX3X at the level of each ribonucleotides of Substrate **$*D_{19}R_1D_4 : D_{24}$** are indicated. **d.** Apparent rate costant (k_{app}) for digestion of DDX3X of Substrate *$\mathbf{D_{39}R_1D_{15} : D_{55}}$ and Substrate *$\mathbf{D_{39}R_1D_{15} : D_{39}8oxoG_1D_{15}}$ are indicated **e.** Coomassie blue stained SDS-PAGE of human recombinant full length DDX5 expressed in *E. coli* and purified from Dyanabeads® His-Tag Isolation & Pulldown beads (Life Technologies). Solid line indicates two portions of the same gel brought next for clarity. **f.** Western blot of human recombinant full length DDX5 of the same fraction shown in panel e. Recombinant DDX5 is detected using an anti-DDX5 polyclonal antibody A300-523A (BETHYL). **g.** DDX5 titration in nuclease assay with Substrate *$\mathbf{D_{39}R_1D_{15} : D_{55}}$. **h.** Nuclease activities of the DDX3X mutants, DDX5 and RNaseH2 proteins assessed on single-strand Substrate *$\mathbf{D_{39}R_1D_{15}}$. All proteins were tested at 2 μM except for RNaseH2 which was tested at 70 nM.

Supplementary Figure S2. Purification of recombinant DDX3X bearing different mutations and assessment of Fen-1 activity. a. Coomassie blue stained SDS-PAGE gels of all recombinant DDX3X mutants expressed in *E. coli* (see Methods for purification details). Solid lines indicate two portions of the same gel brought next for clarity **b.** Comparison between DDX3X and R351A helicase activity. **c.** Fen-1 activity control reaction with the specific flap Substrate $\mathbf{D_{54} : *D_{48} + D_{30}}$ (See Methods for details).

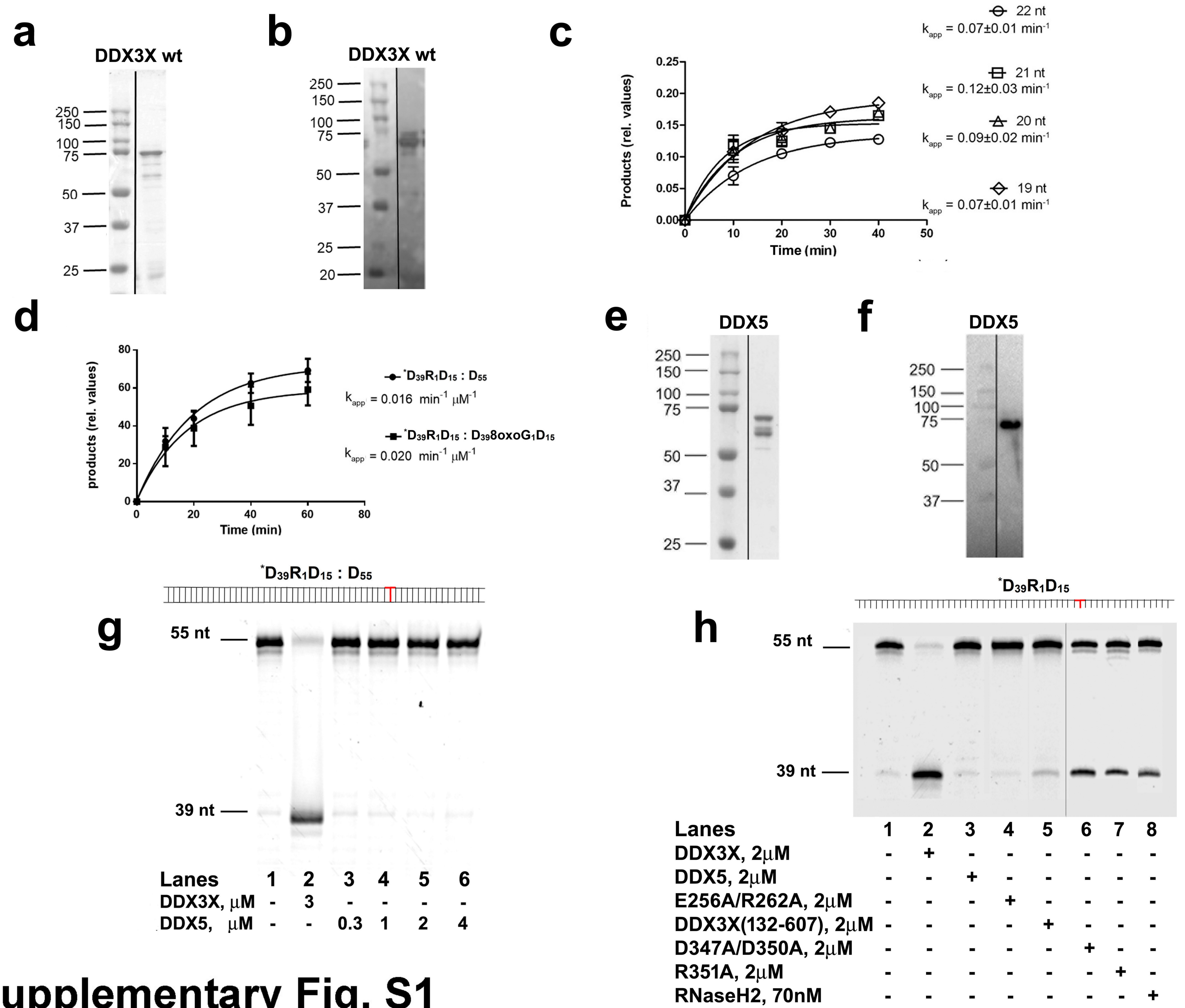

Supplementary Fig. S1

a

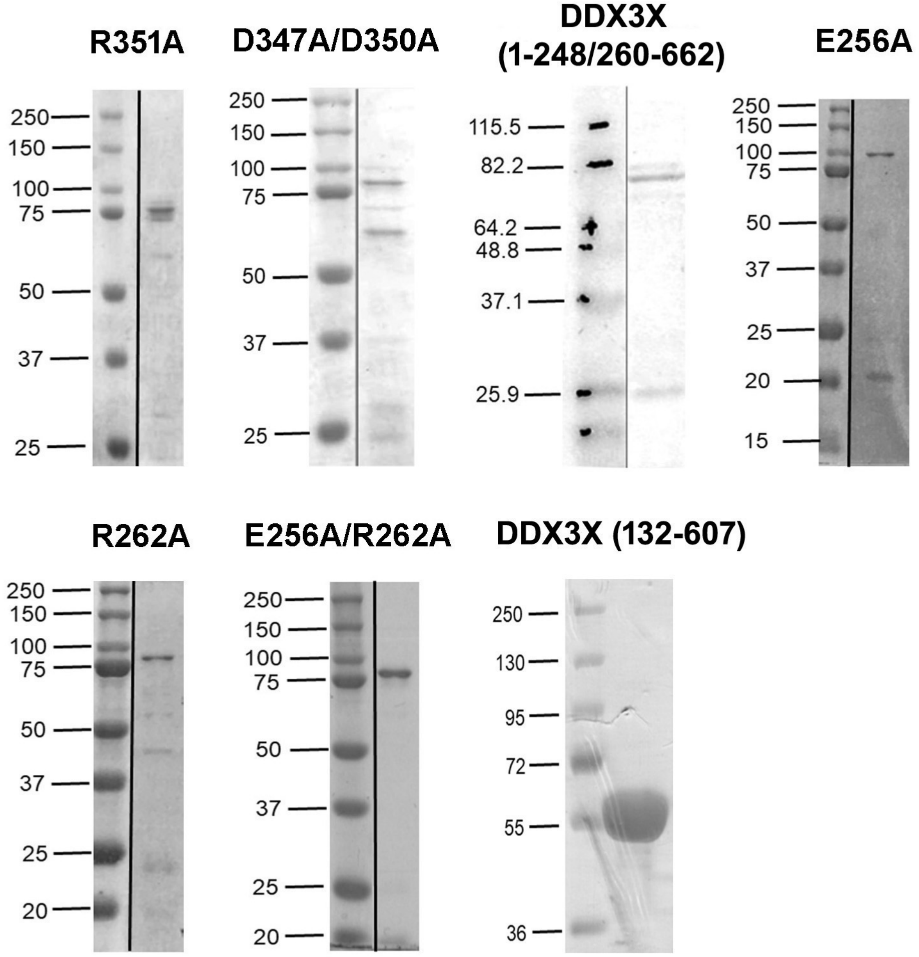

b

Enzyme	Km(RNA) (μM)	kcat (FU[a] · min^{-1})	kcat/Km (μM^{-1}, FU · min^{-1})	-fold reduction[b]
DDX3X wild type	0.31 ± 0.20	2142.14 ± 109.3	6910.13	1
R351A	0.04 ± 0.02	50.02 ± 4.47	1250.5	5.5

c

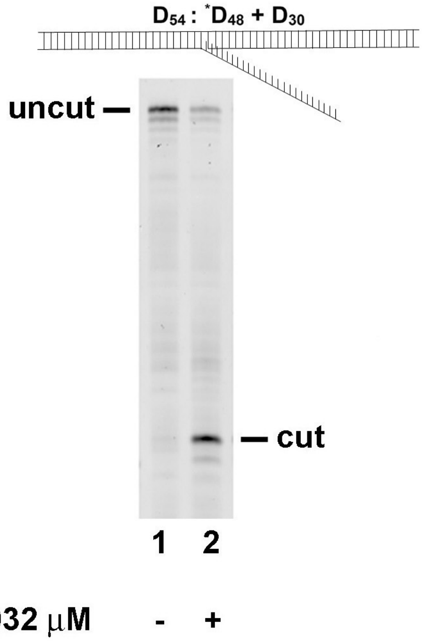

Supplementary Fig. S2
