# Supplementary figures and images for: Correction to ‘Novel alternative ribonucleotide excision repair pathways in human cells by DDX3X and specialized DNA polymerases’
Source: Nucleic Acids Res. 2025 Jun 23;53(12):gkaf578. doi: 10.1093/nar/gkaf578 (PMC12205973; doi:10.1093/nar/gkaf578)

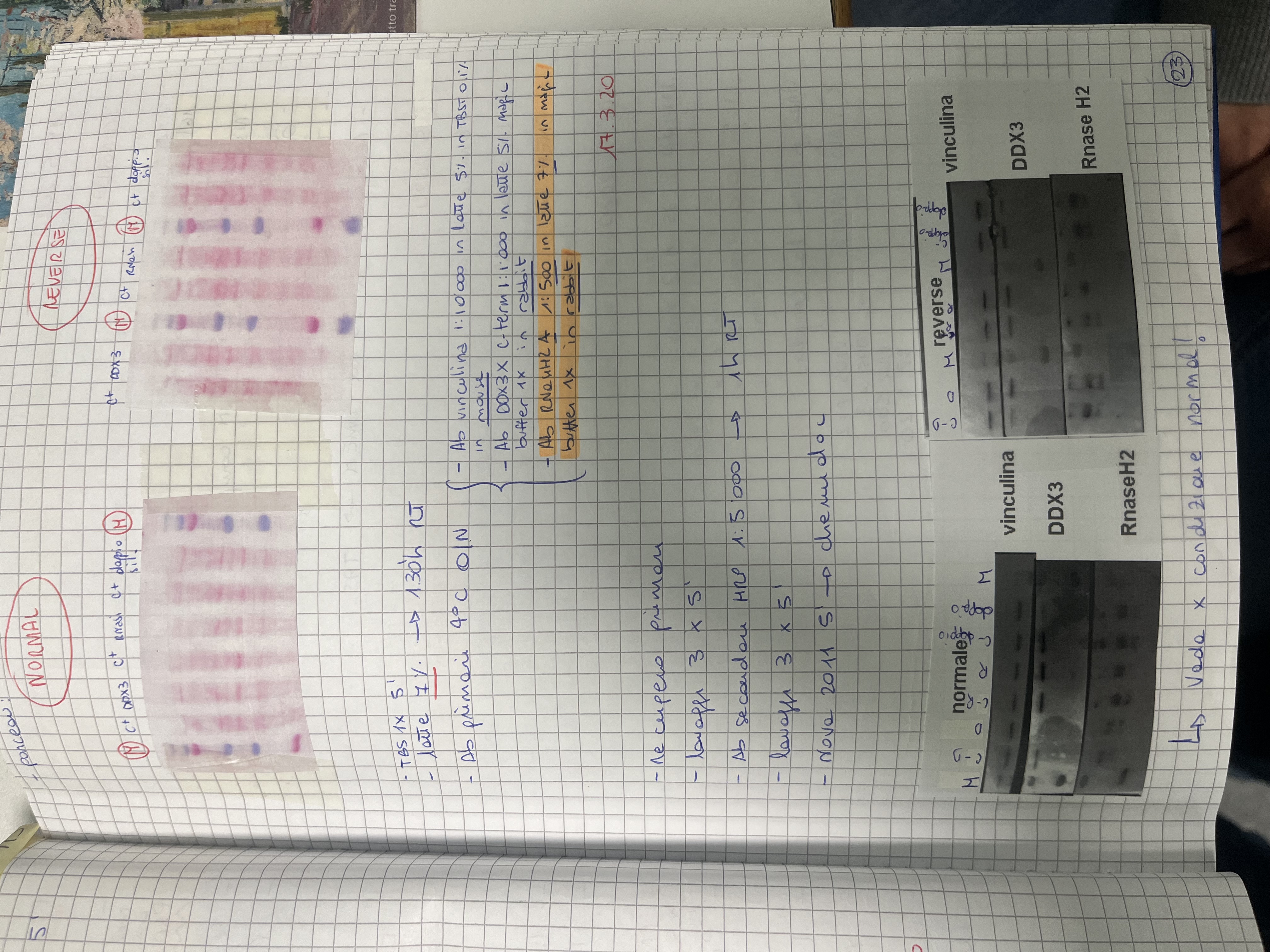

Supplement: gkaf578_Supplemental_Files [file gkaf578_supplemental_files.zip › Lab book exp1.jpg]

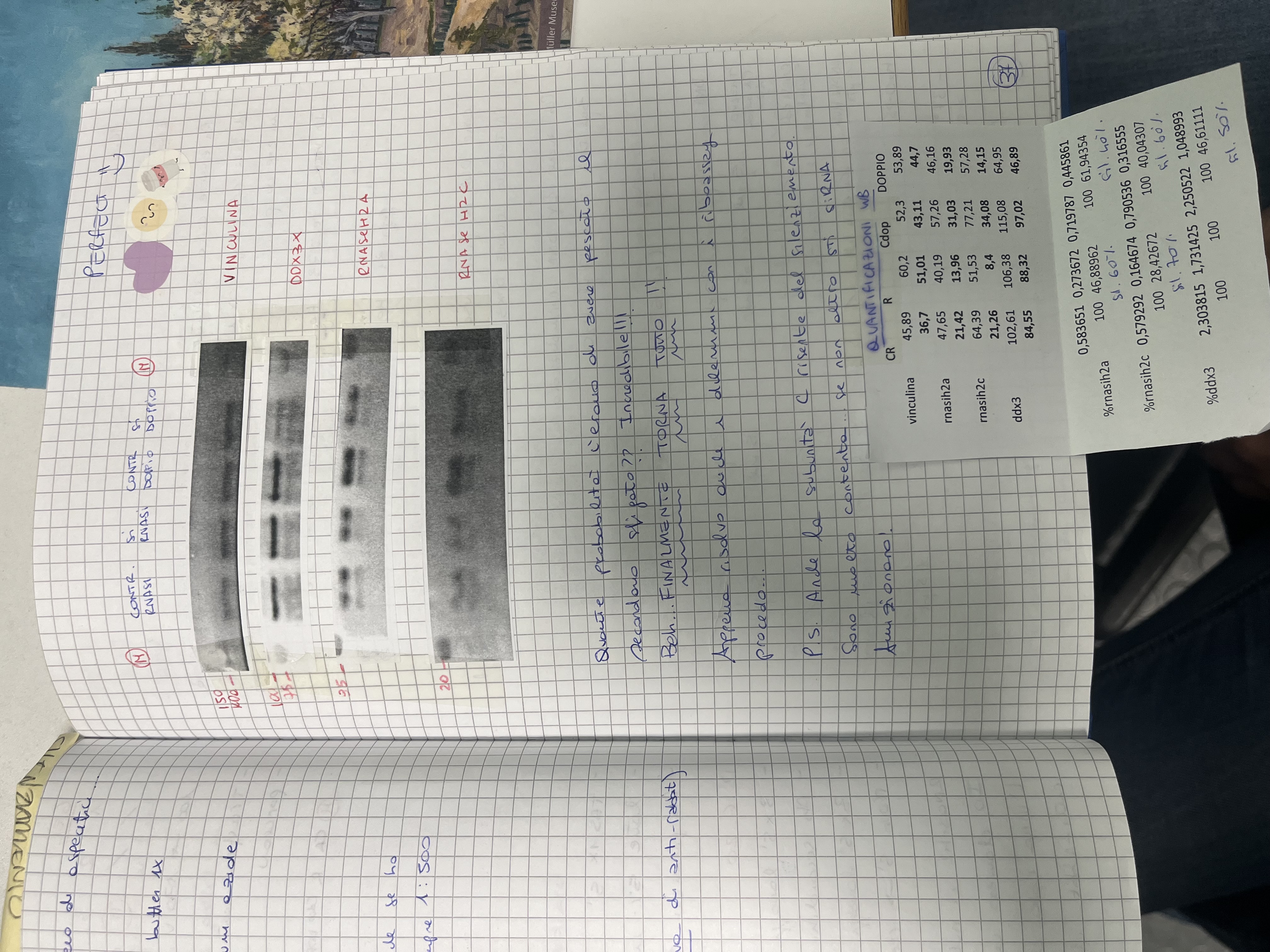

Supplement: gkaf578_Supplemental_Files [file gkaf578_supplemental_files.zip › Lab book exp2.jpg]

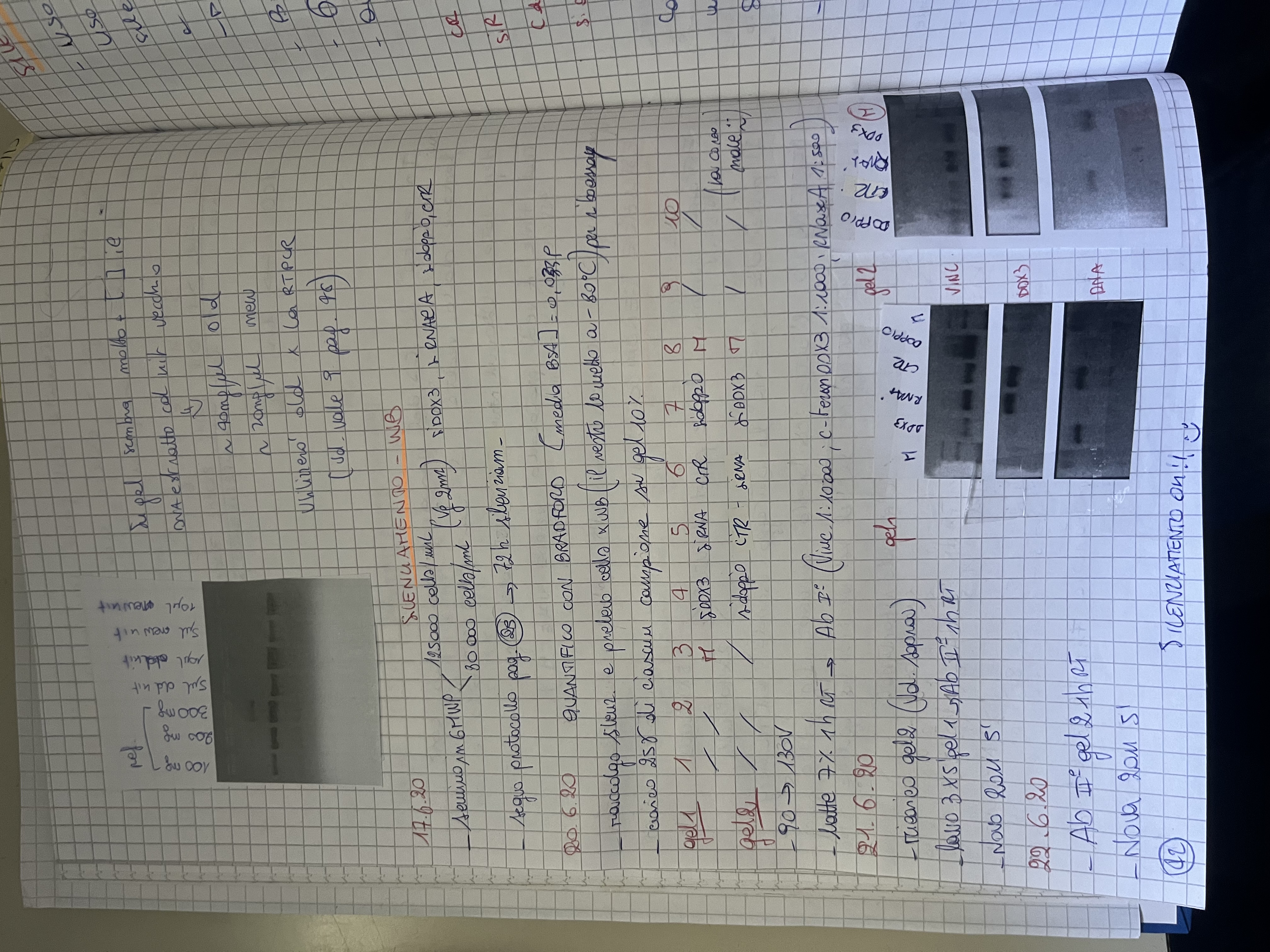

Supplement: gkaf578_Supplemental_Files [file gkaf578_supplemental_files.zip › Lab book exp3-4.jpg]
